# Computational Analysis of *Plasmodium falciparum* DNA Damage Inducible Protein 1 (*Pf*Ddi1): Insights into Binding of Artemisinin and its Derivatives and Implications for Antimalarial Drug Design

**DOI:** 10.1007/s12013-025-01709-2

**Published:** 2025-03-20

**Authors:** Ernest Oduro-Kwateng, Ibrahim Oluwatobi Kehinde, Musab Ali, Kabange Kasumbwe, Vuyisa Mzozoyana, Narasimham L. Parinandi, Mahmoud E. S. Soliman

**Affiliations:** 1 Molecular Bio-Computation and Drug Design Research Group, School of Health Sciences, University of KwaZulu Natal, Westville Campus, Durban, South Africa; 2 Department of Biotechnology and Food Technology, Faculty of Applied Sciences, Durban University of Technology, P.O. Box 1334, Steve Biko Campus, Durban, South Africa; 3 School of Chemistry and Physics, University of KwaZulu-Natal, Westville Campus, Durban, South Africa; 4 Division of Pulmonary, Critical Care, and Sleep Medicine, Department of Medicine, Davis Heart and Lung Research Institute, The Ohio State University Weber Medical Center, Columbus, OH USA

**Keywords:** Human malaria, Artemisinin resistance, *Plasmodium falciparum* DNA-damage-inducible protein 1 (*Pf*Ddi1), In silico modelling

## Abstract

Human malaria remains a global health challenge, with *Plasmodium falciparum* responsible for the most severe cases. Despite global efforts, eradicating malaria has proven difficult, mainly because of the rise in drug resistance, particularly against artemisinin and its derivatives. One possible cause of this resistance is the activation of the unfolded protein response (UPR), which helps maintain cellular balance under stress. In *P. falciparum*, the UPR operates through the ubiquitin-proteasome system (UPS), which involves proteins such as Dsk2, Rad23, and Ddi1. Among these, *Plasmodium falciparum* DNA-damage-inducible protein 1 (*Pf*Ddi1) plays a crucial role in DNA repair and is present throughout the parasite life cycle, making it an attractive drug target. However, there is limited research on *Pf*Ddi1 as a therapeutic target. Recent in vitro studies have indicated that artemisinin (ART) and dihydroartemisinin (DHA) inhibit *Pf*Ddi1 activity. Building on this, we investigated whether ART and its derivatives could serve as inhibitors of *Pf*Ddi1 using computational modeling. Our study included clinically relevant ART derivatives such as artemether (ARM), arteether (AET), artemiside (AMD), and artesunate (ATS). All these compounds showed strong binding to *Pf*Ddi1, with free binding energies ranging from −20.75 kcal/mol for AET to −34.24 kcal/mol for ATS. ARM increased *Pf*Ddi1’s structural rigidity and hydrophobic stability, whereas AMD improved its kinetic stability, resulting in the least residue motion. Unlike AET and AMD, the other ligands destabilize the *Pf*Ddi1 structure. Importantly, three key binding regions—Loop 1 (GLN 266 - ILE 269), Loop 2 (ILE 323 - TYR 326), and Loop 3 (ALA 292 - GLY 294)—were identified as potential targets for new antimalarial drugs against *Pf*Ddi1. This study highlights the potential of ART derivatives as *Pf*Ddi1 inhibitors, paving the way for further experimental validation.

**Graphical Abstract:**

**Figure:**
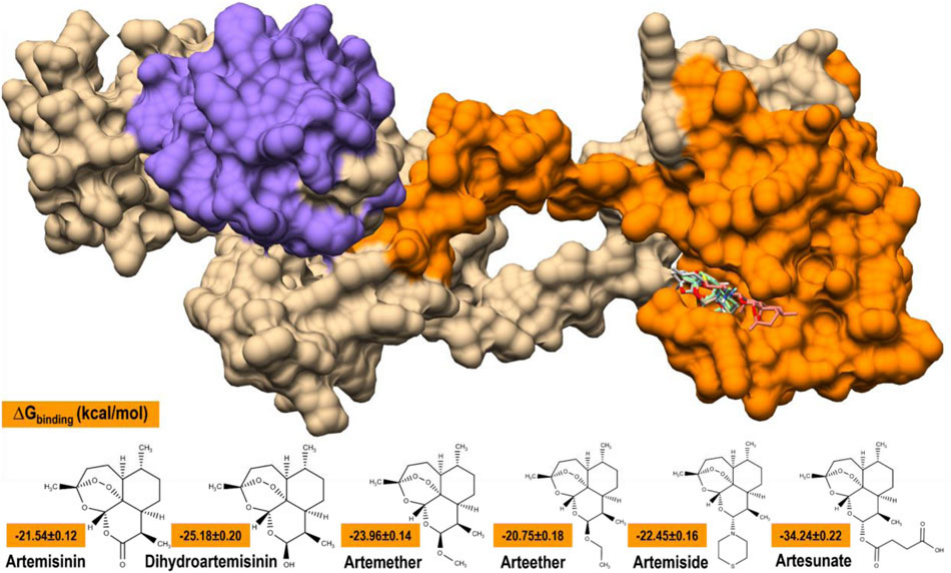
Artemisinin and its derivatives as potential repurposed drugs against *Plasmodium falciparum* DNA-damage-inducible protein 1 *(Pf*Ddi1).

## Introduction

Human malaria infection remains a significant global health concern, with approximately 228 million clinical cases and 405,000 deaths reported in 2018 [1]. The number of cases and deaths continues to be substantial in subsequent years, with an estimated 247 million cases and 619,000 deaths in 2021 and 245 million cases and 625,000 deaths in 2020 [2]. These data underscore the ongoing challenges in meeting the 2030 Malaria Goals. Human malaria is caused by five species of Plasmodium parasites: *Plasmodium falciparum*, *P. vivax*, *P. ovale*, *P. malariae*, and *P. knowlesi*, with *P. falciparum* and *P. vivax* being the most common. *P. falciparum* infection is associated with the most severe form of the disease, while *P. vivax* causes a relapsing form [3]. The infection cycle of *P. falciparum* begins with the injection of sporozoites into the bloodstream through a mosquito bite, which then invades hepatocytes during the liver stage. In liver cells, sporozoites develop into merozoites, which are released into the bloodstream and infect erythrocytes during the blood stage. The parasites undergo various developmental stages, including ring, trophozoite, and schizont stages, within erythrocytes. After completing the 48-h asexual life cycle, the schizonts rupture, releasing daughter merozoites into the plasma to initiate the infection of new erythrocytes [4].

Control of malaria requires a comprehensive approach involving various strategies, such as the use of insecticide-impregnated bed nets, appropriate drug therapies, and vector control measures using insecticides and vaccines. However, the effectiveness of these strategies is challenged by the emergence of resistance to current pharmacotherapies and commonly used insecticides [5]. Historically, antimalarial drugs, such as chloroquine and sulfadoxine/pyrimethamine, have been crucial for malaria control, but resistance to these drugs has limited their effectiveness [6, 7]. Currently, artemisinin and its derivatives (ARTs) are essential components for frontline malaria treatment, particularly for *Plasmodium falciparum* infections [8]. Artemisinin-based combination therapies (ACTs), which combine artemisinin with longer-lasting partner drugs, such as piperaquine, lumefantrine, or mefloquine, are widely used for their efficacy and reduced risk of resistance development [9, 10].

The emergence and spread of resistance to artemisinin and its derivatives poses a significant threat to global malaria elimination efforts [11, 12]. The *P. falciparum* Kelch 13 gene (*Pf*Kelch13) has been identified as a major marker for artemisinin resistance [13]. Two other proposed mechanisms of artemisinin resistance include activation of the unfolded protein response (UPR) and dysregulation of *P. falciparum* phosphatidylinositol 3-kinase (*Pf*PI3K) [14]. Additionally, recent research suggests that host immunity may also play a role in artemisinin resistance, posing a unique challenge to malaria elimination [15, 16]. Furthermore, studies have highlighted the broad cellular damage caused by artemisinin and derivatives, including protein insults and DNA damage, mediated by reactive oxygen species (ROS) to aggravate resistance [17, 18]. This damage activates pathways, such as the unfolded protein response (UPR), including the ubiquitin-proteasome system (UPS), which is crucial for maintaining cellular homeostasis [19]. The UPS, which is responsible for clearing unwanted or misfolded proteins, has been implicated in the development of malarial parasites, suggesting its potential as a drug target for malaria treatment [20].

**Fig. 1:**
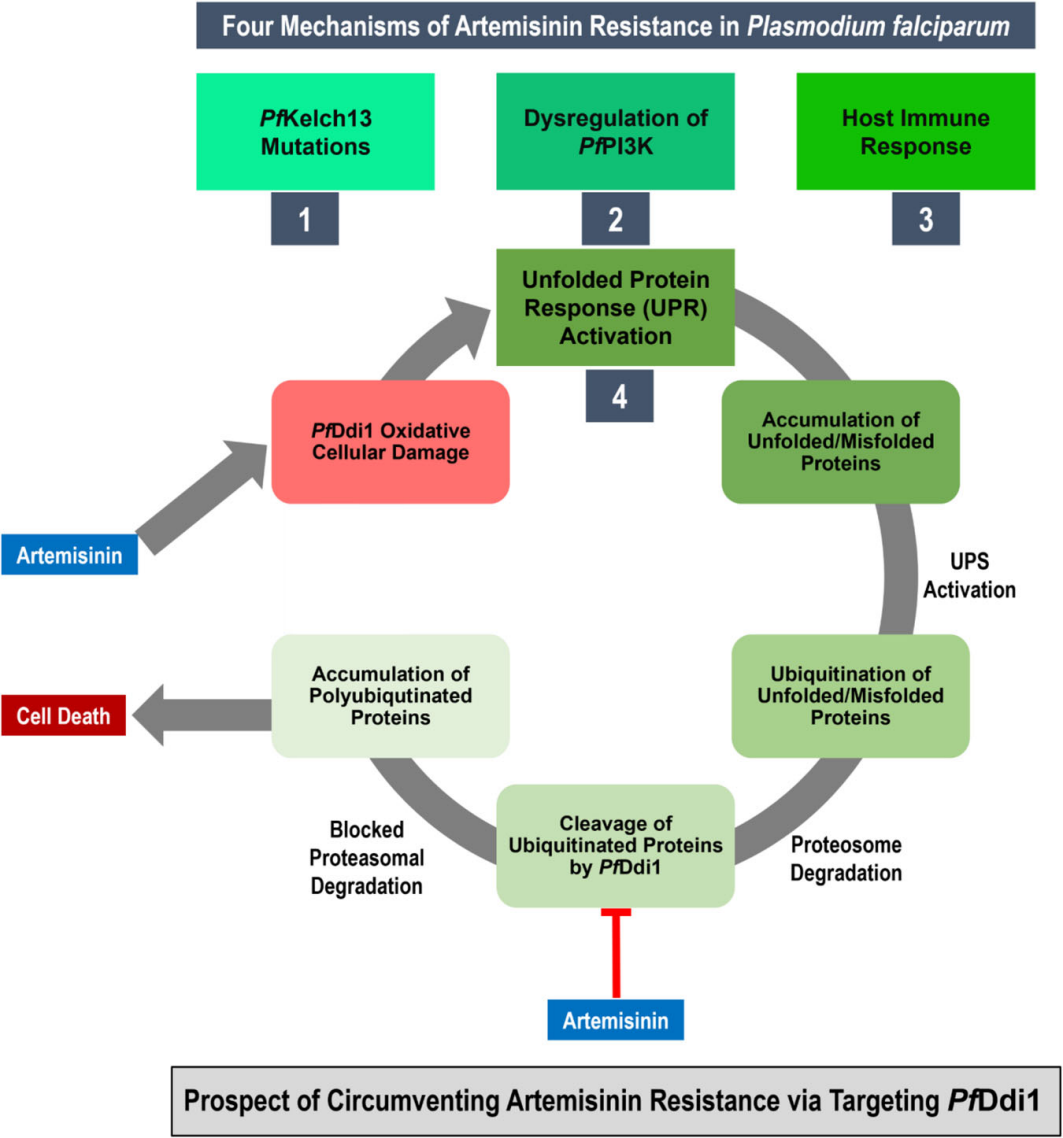
Schematic diagram highlighting the physiological role of the unfolded protein response (UPR) machinery in the survival of *Plasmodium falciparum* and its resistance to artemisinin. In addition, the potential of repurposing *Pf*Ddi1 as a druggable target to alleviate artemisinin resistance is proposed

The ubiquitin-proteasome system (UPS) is a tightly regulated pathway that targets proteins for proteasomal degradation via ubiquitin attachment. Proteins such as Rad23, Dsk2, and Ddi1 play crucial roles in facilitating recognition, ubiquitination, and degradation of specific protein substrates within the cell [21]. These proteins contain ubiquitin-like (UBL) and/or ubiquitin-associated (UBA) domains, enabling their interaction with both the proteasome and ubiquitin chains on the target protein [22]. Among these proteins, *Plasmodium falciparum* Ddi1 (*Pf*Ddi1) stands out because of its essential role in parasite survival, unique protozoan family retroviral protease (RVP) domain structure, and specific enzymatic activity in cleaving ubiquitinated substrates [20]. Notably, the protozoan family RVP has a highly conserved catalytic motif, presenting an attractive drug target for treating infections caused by other trypanosomatid parasites, such as Leishmania [20]. *Pf*Ddi1 is synthesized across all major life stages of the parasite and is associated with chromatin and DNA-protein crosslinks, indicating its importance in DNA repair mechanisms [23]. Gene knockout experiments may have demonstrated the role of *Pf*Ddi1 in mediating ART resistance. Onchieku et al. [20] used *Saccharomyces cerevisiae* cells that had a knockout of the Ddi1 gene. These knockout cells were more susceptible to ART treatment, indicating that the absence of *Pf*Ddi1 compromised their ability to withstand ART pressure. Furthermore, the study found that the introduction of *Pf*Ddi1 into these knockout cells restored their resistance to ART, suggesting that *Pf*Ddi1 plays a crucial role in the cellular response to ART and may be involved in the mechanisms that confer resistance in *P. falciparum*. Furthermore, the study quantitatively assessed the inhibition of *Pf*Ddi1 by ART, showing significant inhibition percentages (71.4% for retropepsin substrates and 65.9% for proteasome substrates) [20]. Therefore, targeting *Pf*Ddi1 with inhibitors could potentially circumvent the existing resistance mechanisms and offer a promising avenue for the development of effective antimalarial therapies [23, 24] (Fig. 1).

To date, research on the antimalarial potential of *Pf*Ddi1 is limited. Onchieku et al. [20] demonstrated that *Pf*Ddi1 binds to both artemisinin and dihydroartemisinin in the retroviral protease (RVP) domain. This suggests that artemisinin and its derivatives could serve as potential drugs or probes for *Pf*Ddi1. To further explore this potential, we investigated the antimalarial potency of other artemisinin derivatives, including artemether (ARM), arteether (AET), artemiside (AMD), and artesunate (ATS), as well as dihydroartemisinin (DHA) and artemisinin (ART), using computational methods. These drugs were selected based on their therapeutic relevance in preclinical and mainstream *P. falciparum* malaria therapies. For example, artesunate, artemether, and dihydroartemisinin are available in combination regimens with amodiaquine [25], lumefantrine [26], and piperaquine [27], respectively as approved oral treatments for acute, uncomplicated malaria. Additionally, arteether is administered intramuscularly to treat severe, complicated malarial infections [28]. Artemiside, on the other hand, is a novel compound that exhibits potent antimalarial effects against both asexual and sexual stages of *P. falciparum* strains in vitro [29]. The objective of our study was to elucidate the molecular mechanisms underlying the inhibition of *Pf*Ddi1 and provide further evidence supporting artemisinin and its derivatives as potential inhibitors of *Pf*Ddi1. Our findings on the structural conformational dynamics offer insights for future studies to design more effective antimalarial drugs targeting *Pf*Ddi1.

## Methodology

### Residue Sequence Retrieval, Structure Selection, and Validation

**Fig. 2:**
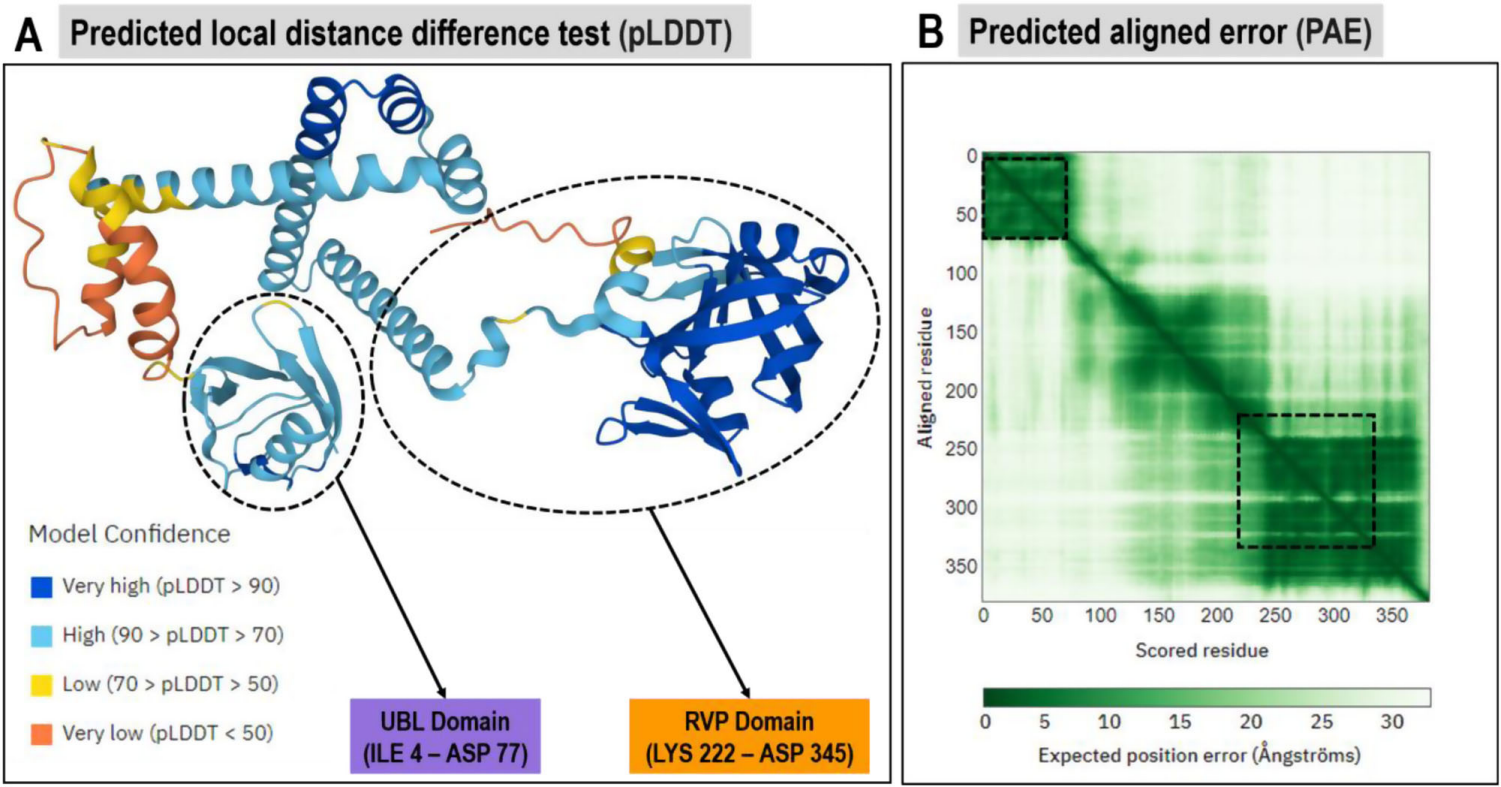
Validation of the predicted structure of full-length *Pf*Ddi1 using **A** predicted local distance difference test (pLDDT) and **B** predicted aligned error (PAE). The UBL (medium purple) and RVP (orange) domains are highlighted in both evaluation tools

To the best of our knowledge, a full-length 3D crystal structure of *Pf*Ddi1 is not yet available [20]. Therefore, the residue sequence of PfDdi1 (ID: Q8IM03) from 3D7 strain was retrieved from the Universal Protein Resource (UniProt) (https://www.uniprot.org/). This sequence served as the basis for structural homology searches using the BLASTp program of NCBI (https://www.ncbi.nlm.nih.gov/). The search identified the template with accession code XP_001348263, which exhibited 100% sequence identity, 100% query coverage, and an E-value of 0, making it suitable for homology modeling studies. To build a reliable model, we evaluated five templates (Q8IM03, 7EFY, 4RGH, 5YS4, and 4Z2Z) based on sequence identity, Global Model Quality Estimate (GMQE), and residue coverage (Table 1). Q8IM03 demonstrated 100% sequence identity, full-length residue coverage, and the most optimal GMQE value (0.80), distinguishing it as the best fit for modeling. This structure, generated using AlphaFold v2.0, was selected for further validation. The Q8IM03 structure was validated using the predicted Local Distance Difference Test (pLDDT) scores and Predicted Aligned Error (PAE) metrics from AlphaFold. The pLDDT scores were >90 for residues in the RVP domain and 70–90 for most UBL residues, reflecting a high confidence in the predicted local residue positions (Fig. 2A). PAE scores showed high confidence in the interdomain accuracy of the RVP domain (Fig. 2B), confirming the structural reliability of Q8IM03.

**Table 1:** Sequence characteristic features of *Pf*Ddi1 templates

*PfDdi1* Template	Residue Range (aa)	Organism	GMQE value	Sequence Identity (%)	Sequence Similarity (%)	Sequence Coverage
Q8IM03	1–382	*Plasmodium falciparum*	0.80	100.00	0.61	1.00
7EFY	243–364	*Cryptosporidium hominis*	0.23	61.29	0.49	0.32
4RGH	242–366	*Homo sapiens*	0.21	49.28	0.45	0.36
5YS4	239–366	*Leishmania major*	0.23	48.44	0.44	0.34
4Z2Z	231–367	*Saccharomyces cerevisiae*	0.22	46.10	0.42	0.37

AlphaFold v2.0, an advanced deep-learning AI system, predicts protein structures with atomic precision by leveraging neural networks to analyze amino acid sequences. Its performance surpasses that of traditional methods such as homology modeling by providing insights into residue flexibility and thermodynamic behavior [30, 31]. The AlphaFold Protein Structure Database (https://alphafold.ebi.ac.uk), developed by DeepMind and EMBL-EBI, offers the Q8IM03 model as an open-source, high-resolution protein structure prediction tool, ensuring a credible basis for the structural studies in this work [32, 33].

### Binding Pocket Identification and System Preparation

#### Binding Pocket Identification and Validation

**Fig. 3:**
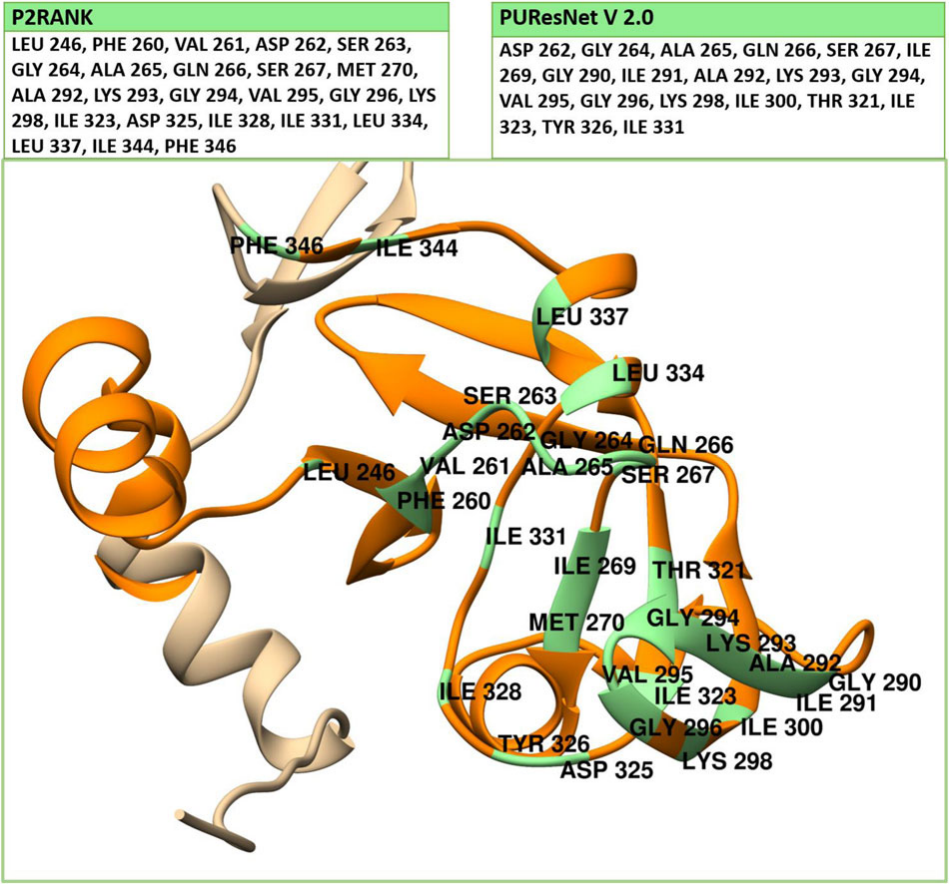
Active-site binding pocket of *Pf*Ddi1 RVP (orange) showing the position of the key residues (light green), identified using P2Rank and PUResNet

Identification and validation of a protein-binding pocket from experimental data is a crucial step in understanding protein-ligand interactions, which is essential for drug discovery and design. In this study, the active binding pocket of *Pf*Ddi1 was identified and validated using P2Rank and PUResNet protocols. These advanced tools leverage machine learning and deep neural networks to accurately predict binding sites and provide a robust framework for structural analysis [34]. Using the P2Rank protocol (http://prankweb.cz/), the ligandability of protein surface points based on local geometric and physicochemical properties was calculated. High scoring points were clustered and ranked, and the binding site was defined with strong predictive confidence [35]. Similarly, PUResNet (https://nsclbio.jbnu.ac.kr/tools/jmol/) applies a deep residual neural network to treat the *Pf*Ddi1 structure as a 3D input, generating voxel-level binding site predictions [36]. These template-free approaches were suitable for validating the active binding pocket of *Pf*Ddi1, defined to involve LEU 246, PHE 260, VAL 261, ASP 262, SER 263, GLY 264, ALA 265, GLN 266, SER 267, ILE 269, MET 270, GLY 290, ILE 291, ALA 292, LYS 293, GLY 294, VAL 295, GLY 296, LYS 298, ILE 300, THR 321, ILE 323, ASP 325, TYR 326, ILE 328, ILE 331, LEU 334, LEU 337, ILE 344, and PHE 346 (Fig. 3). The results obtained corroborate the experimental data from Onchieku et al. [20], which indicated residues VAL 243, PHE 244, MET 245, LEU 246, PHE 260, VAL 261, ASP 262, SER 263, and GLY 264 as RVP-active interacting residues.

#### Computational Preparation of Artemisinin and its Derivatives

**Fig. 4:**
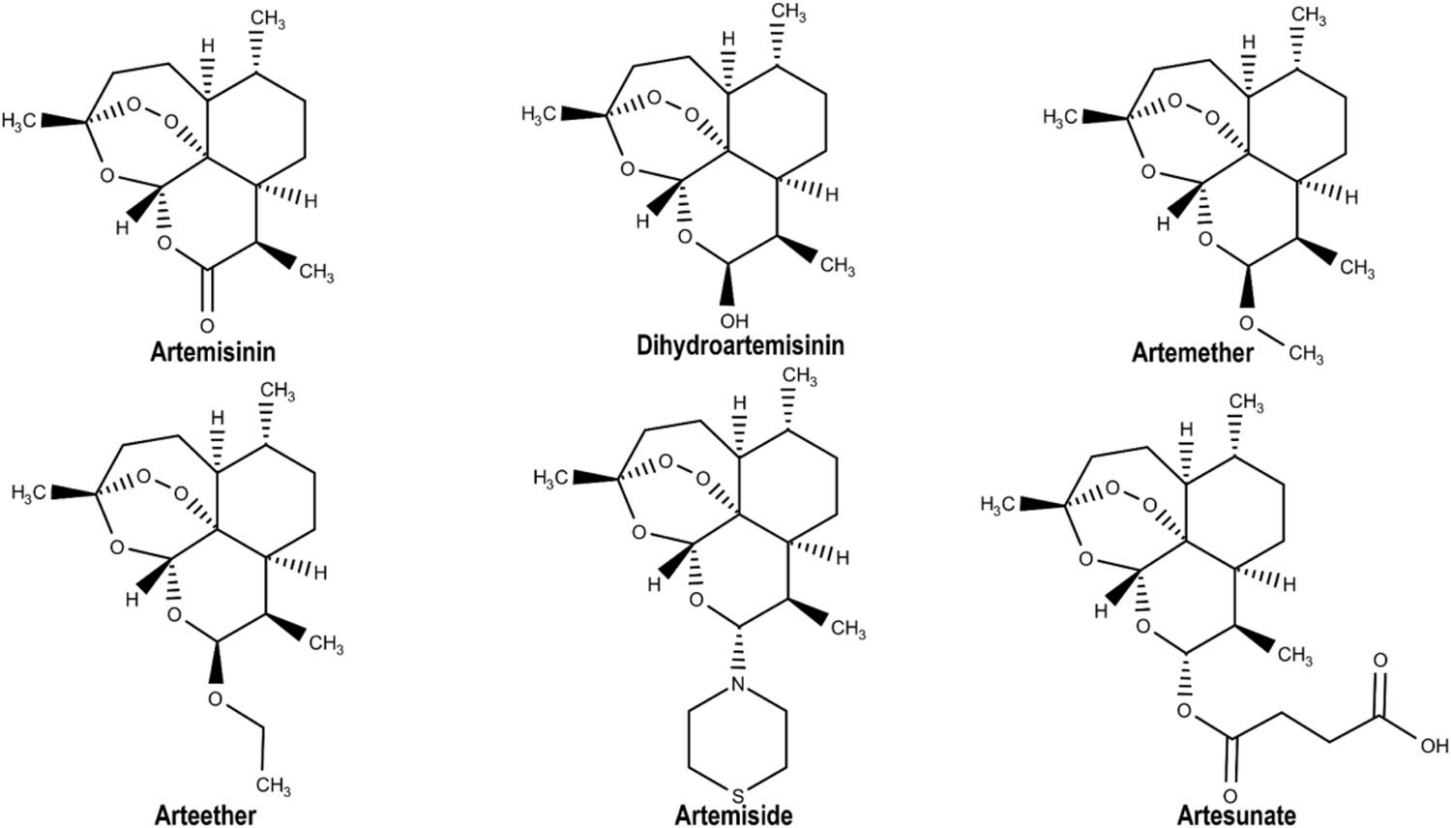
2D representation of the coordinates of artemisinin (ART) and its derivatives

Artemisinin and dihydroartemisinin have been reported to inhibit *Pf*Ddi1 in vitro [20]. Therefore, we prepared these inhibitors in addition to other artemisinin analogs for molecular modelling studies. The 3D coordinates of artemisinin (ART), dihydroartemisinin (DHA), artemether (ARM), arteether (AET), artemiside (AMD), and artesunate (ATS), with compound IDs of 68827, 3000518, 68911, 3000469, 49773910, and 6917864, respectively were retrieved from PubChem (https://pubchem.ncbi.nlm.nih.gov/). The ligands were optimized using Molegro Molecular Viewer (MMV). Bond angles and hybridization states were corrected where necessary. UCSF Chimera Tools were then used to minimize the energies of *Pf*Ddi1 structure at 100 steepest descent steps, 0.02 steepest descent step size (Å), 10 conjugate gradient steps, and 0.02 conjugate gradient step size (Å) of 10 update intervals, and Gasteiger charges were added via the ANTECHAMBER tool [37]. After neutralizing the system by adding hydrogen atoms, we used the H++ server (http://newbiophysics.cs.vt.edu/H++/) to predict the protonation state of the protein at physiological pH [38]. However, consistent results were obtained using the AMBER force field, which employs a similar methodology. Figure 4 shows the 2D chemical structures of ART and its derivatives.

### Binding Score Calculations

Binding score calculations were performed using AutoDock Vina within the UCSF Chimera workspace [39]. AutoDock Vina employs the lamarckian genetic algorithm coupled with advanced gradient optimization techniques to achieve precise docking results. Utilizing multithreading capabilities, AutoDock Vina enhances both the speed and accuracy in the clustering of grid map results. Renowned for its efficiency and accuracy, AutoDock Vina is a leading molecular docking program that consistently outperforms other widely adopted softwares [40]. In our study, molecular docking was performed with a focus on the defined active site of *Pf*Ddi1 RVP to enhance precision and minimize the potential for false-positive interactions with the ligands. The Autodock Tools interface facilitated the specification of the grid volume within the active site by employing a 5 Å spacing in the x-, y-, and z-dimensions. The grid center coordinates were set at (9.83272 × −0.425104 × −10.38550), with dimensions defined as (24.29350 × 30.43140 × 28.59450), respectively. Docking poses were visualized within the Chimera workspace, allowing direct observation and analysis. Docking scores were determined based on the lowest negative values obtained, ensuring comprehensive evaluation of the molecular docking results.

### Molecular Dynamics (MD) Simulation

Preparation of *Pf*Ddi1 and its inhibitors for MD simulations was performed using UCSF Chimera Tools, which involved the removal of explicit hydrogen atoms from the protein and the addition of AMBER charges to the inhibitors. MD simulations were conducted using the Particle Mesh Ewald Molecular Dynamics (PMEMD) Compute Unified Device Architecture (CUDA) single graphic processor unit (GPU) in the AMBER 18 package [41]. The FF14SB AMBER force field was utilized to parameterize the proteins, whereas the ANTECHAMBER protocol added partial charges to the inhibitors through the application of restrained electrostatic potential (RESP) and GENERAL AMBER Force Field (GAFF) procedures. Subsequently, the LEAP module neutralized and solvated all systems by adding hydrogen atoms, sodium ions, and chloride counter ions. Atomic solvation was performed in an orthorhombic TIP3P box with 10 Å water molecules. The pdb4amber command modified the *Pf*Ddi1 protein system topologies before executing the LEAP protocol. Partial minimization (2500 steps) with a 500 kcal/mol restraint potential and full minimization (5000 steps) without conjugate energy restraint were performed [42]. Following the preparation, all systems were heated from 0 to 300 K for 50 ps in a canonical ensemble (NVT) using a Langevin thermostat and a harmonic potential restraint of 10 kcal/mol Å. The SHAKE algorithm was employed for hydrogen-bond constraints, and the pressure was maintained at 1 bar using a Barendsen-Barostat. MD simulations were conducted for 200 ns in an isothermal-isobaric (NPT) ensemble at a time scale of 2 fs, and the temperature was maintained at 300 K using a Langevin thermostat and a pressure of 1 bar. The coordinates were saved every 1 ps and analyzed using the CPTRAJ and PTRAJ modules of the AMBER18 GPU. Post-MD analyses included root mean square deviation (RMSD), root mean square fluctuation (RMSF), radius of gyration (ROG), solvent-accessible surface area (SASA), Dynamic Cross-Correlation Matrix (DCCM), and Principal Component Analysis (PCA). Visualization and structural analyses were performed using graphical software packages, such as VMD, UCSF Chimera, and Discovery Studio 2021, while Origin software was used for data plotting [43].

### Binding Free Energy (BFE) Computations

The molecular mechanics/generalized Born surface area (MM/GBSA) protocol was used to estimate the free energy of binding (BFE) between artemisinin and its derivatives to *Pf*Ddi1 within the bound complexes [42, 43]. This approach combines molecular mechanics calculations with the generalized Born (GB) dielectric continuum solvent model and surface area (SA) terms to evaluate the BFE. Molecular mechanics calculations involve internal energies, van der Waals interactions, electrostatic interactions, and other molecular forces [44]. The GB model computes the polar solvation free energy by considering the Born radii of the atoms and their pairwise interactions. Conversely, the SA method quantifies the reduction in hydrophobic interactions upon binding by computing the buried surface area (BSA) during complex formation, correlating the nonpolar solvation energy with the surface area of the protein-ligand interface using a water probe radius of 1.4 Å and surface tension constant (γ) set at 0.0072 kcal/mol Å^2^. BFE calculations were estimated using 50,000 complex frames due to the computational cost of calculating the change in conformational energy for large frames in a normal node [42].

The formula for BFE (ΔG) computation is as follows:

$${\Delta G}_{{bind}}={G}_{{complex}}-{G}_{{receptor}}-{G}_{{ligand}}$$

$${\Delta G}_{{bind}}={E}_{{gas}}+{G}_{{sol}}-T\Delta S$$

$${E}_{{gas}}={E}_{\mathrm{int}}+{E}_{{vdw}}+{E}_{{ele}}$$

$${G}_{{sol}}={G}_{{GB}}+{G}_{{SA}}$$

$${G}_{{SA}}=\gamma {SASA}$$

Where:

E_gas_ represents the gas-phase summation comprising the internal energy (E_int_), Coulomb energy (E_ele_), and van der Waals energy (E_vdw_). G_sol_ denotes the free solvation energy, which is the sum of the generalized Born (GB) energy (G_GB_) and the surface area (SA) energy (G_SA_), and TΔS represents the total interaction entropy. E_gas_ was computed using the AMBER FF14SB force field, while G_sol_ was determined from the energy contributions of polar and nonpolar states.

### Per-Residue Energy Decomposition (PRED) Analysis

The MM/GBSA method implemented in AMBER 18 was employed to calculate the energy contribution of each residue to the total binding free energy (BFE) of the *Pf*Ddi1-bound systems across 50,000 snapshot frames [42, 43].

### Receptor – Ligand Interaction Analysis

Discovery Studio Visualizer 2021 Client served as the primary tool for analyzing residue-ligand interaction network, showcasing the non-covalent bond types between *Pf*Ddi1 and its inhibitors. Post-MD trajectory snapshots were visualized to elucidate the interaction networks for all bound complexes, providing insights into their interactions and highlighting the differences between them.

## Results and Discussion

### Binding Modes of Artemisinin and Derivatives: High Binding Affinity for RVP Domain

**Fig. 5:**
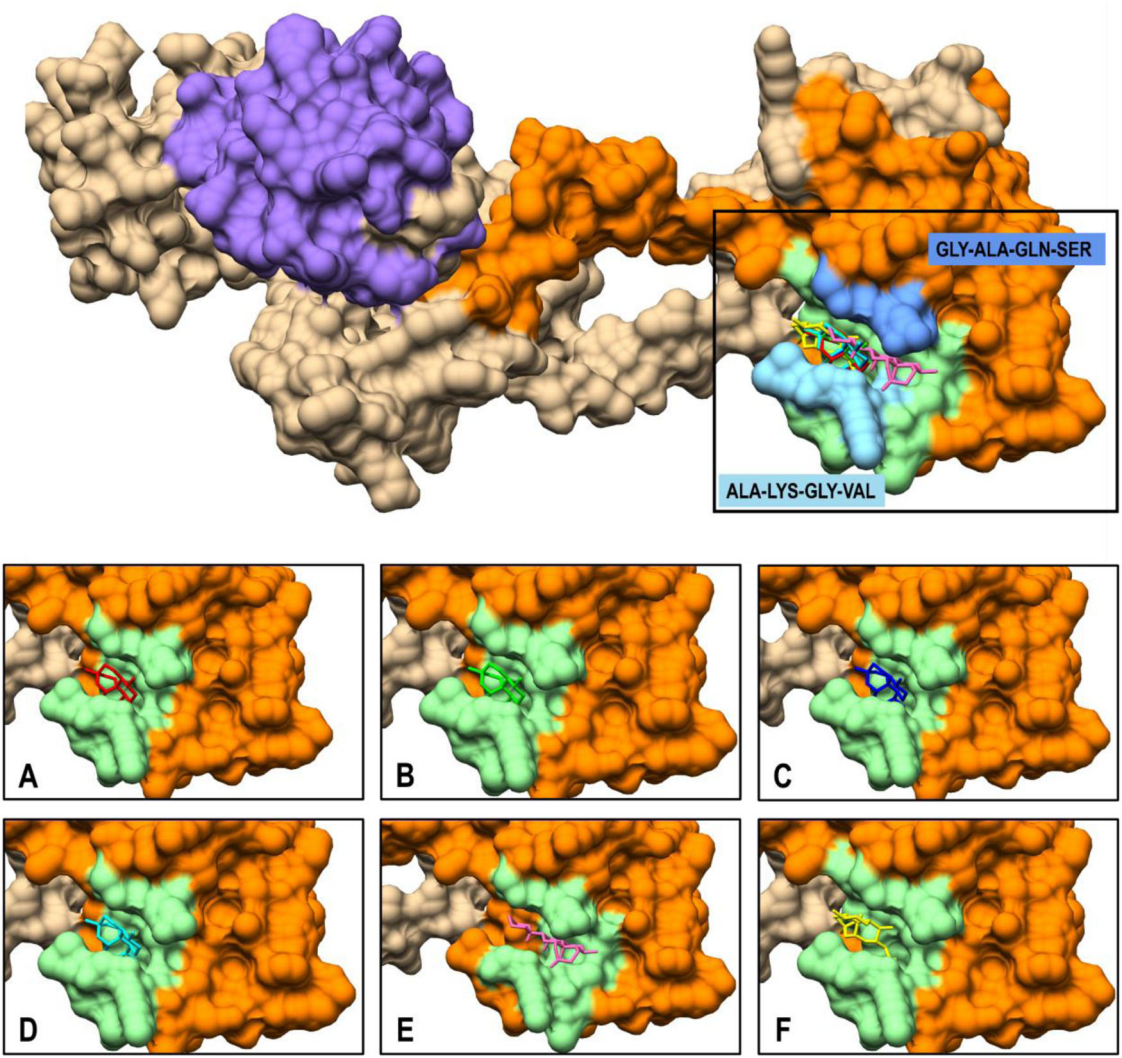
3D solid structure of full-length *Pf*Ddi1 (tan) showing the functional UBL domain (medium purple) and RVP domain (orange) harboring the docking pose of **A** ART (red), **B** DHA (green), **C** ARM (blue), **D** AET (cyan), **E** AMD (pink), and **F** ATS (yellow). Each ligand consistently interacted with GLY-ALA-GLN-SER (264–267) (cornflower blue) and ALA-LYS-GLY-VAL (292–295) (sky blue) RVP motifs

Artemisinin has been found to disrupt *Plasmodium falciparum*’s survival strategy after cellular damages by blocking its DNA-damage-inducible protein 1 (Ddi1) enzyme at the RVP domain [20]. To determine the degree of binding affinity, we performed molecular docking studies on *Plasmodium falciparum* Ddi1 (*Pf*Ddi1) using artemisinin (ART) and its derivatives, dihydroartemisinin (DHA), artemether (ARM), arteether (AET), artemiside (AMD), and artesunate (ATS). The docking scores for ART, DHA, ARM, AET, AMD, and ATS were −7.2, −7.4, −6.4, −6.4, −6.8, and −7.0 kcal/mol, showing that all the ligands are effective against *Pf*Ddi1. DHA exhibited the highest binding affinity for *Pf*Ddi1, whereas ARM and AET had the lowest binding affinities. Docking calculations revealed that RVP interaction residues (ASP 262, GLY 264, ALA 265, GLN 266, SER 267, ILE 269, ALA 292, LYS 293, GLY 294, VAL 295, THR 297, ILE 323, TYR 326, ILE 328, and ILE 331) were highly conserved among all ligands, with unique interaction residues: PHE 260 for ART and ILE 291, ILE 300, and THR 321 for AMD (Table 2). Furthermore, all the ligands were found to bind similarly in an open deep groove flanked by loops, GLY-ALA-GLN-SER (264–267) and ALA-LYS-GLY-VAL (292–295) (Fig. 5). Our findings highlight *Pf*Ddi1 RVP as an appealing target for antimalarial therapy, particularly the identified loops as potential weak regions for drug development.

**Table 2:** Compound IDs of *Pf*Ddi1 ligands, their docking scores expressed in kcal/mol, and their respective active-site residues within 5 Å in the RVP domain

*Pf*Ddi1 Ligand	Compound ID	Docking score (kcal/mol)	Interacting Active-site Residues
Artemisinin (ART)	CID 68827	−7.2	PHE 260, **ASP 262,** **GLY 264,** **ALA 265,** **GLN 266,** **SER 267,** **ILE 269,** **ALA 292,** **LYS 293,** **GLY 294,** **VAL 295**, GLY 296, THR 297, LYS 298, ILE 323, TYR 326, ILE 328, ILE 331
Dihydroartemisinin (DHA)	CID 3000518	−7.4	PHE 260, **ASP 262,** **GLY 264,** **ALA 265,** **GLN 266,** **SER 267,** **ILE 269,** **ALA 292,** **LYS 293,** **GLY 294,** **VAL 295**, GLY 296, THR 297, LYS 298, ILE 323, TYR 326, ILE 328, ILE 331
Artemether (ARM)	CID 68911	−6.4	PHE 260, **ASP 262,** **GLY 264,** **ALA 265,** **GLN 266,** **SER 267,** **ILE 269,** **ALA 292,** **LYS 293,** **GLY 294,** **VAL 295**, GLY 296, THR 297, LYS 298, ILE 323, TYR 326, ILE 328, ILE 331
Arteether (AET)	CID 3000469	−6.4	PHE 260, **ASP 262,** **GLY 264,** **ALA 265,** **GLN 266,** **SER 267,** **ILE 269,** **ALA 292,** **LYS 293,** **GLY 294,** **VAL 295**, GLY 296, THR 297, LYS 298, ILE 323, TYR 326, ILE 328, ILE 331
Artemiside (AMD)	CID 49773910	−6.8	**ASP 262,** **GLY 264,** **ALA 265,** **GLN 266,** **SER 267,** **ILE 269**, ILE 291, **ALA 292,** **LYS 293,** **GLY 294,** **VAL 295**, ILE 300, THR 321, ILE 323, TYR 326, ILE 331
Artesunate (ATS)	CID 6917864	−7.0	PHE 260, **ASP 262,** **GLY 264,** **ALA 265,** **GLN 266,** **SER 267,** **ILE 269,** **ALA 292,** **LYS 293,** **GLY 294,** **VAL 295**, GLY 296, THR 297, LYS 298, ILE 323, TYR 326, ILE 328, ILE 331

The common residues involved in the interactions at the active site across all the *Pf*Ddi1-ligand complexes have been highlighted

We sought to investigate whether structural variations among ART derivatives had a statistically significant impact on binding affinity (Tables S1 and S2). A One-Way ANOVA was performed, and the results indicate a highly significant difference between the ligand groups (P-value < 6.6718 × 10^−177^). The large F-value (8.65383 × 10^29^) further confirmed that there were significant differences in the binding scores between the ART derivatives (Table S3). To determine which specific ligand pairs differed significantly in their docking scores, Tukey’s post hoc test was conducted. The results provide a detailed comparison of the mean differences between pairs of ligands, their corresponding Q-values, and P-values. Significant differences were found in most ligand pairs, as indicated by p-values of <0.05. However, the ARM vs. AET pair yielded a mean difference of 0 with a p-value of 1, indicating no significant difference in binding affinity between these two ligands given their nearly identical structural similarity (Table S4). While the inclusion of One-Way ANOVA and Tukey’s comparison test in molecular docking studies is not a standard practice, their application in this context provides a statistical evaluation of the differences in binding affinities among artemisinin derivatives docked to the *Pf*Ddi1 active site. It is important to emphasize that docking scores alone do not capture the full complexity of ligand-protein interactions without further corroboration from advanced computational techniques, such as free binding energy calculations, as detailed in this study. Additionally, experimental validation is necessary to further confirm the computational findings and understand the biological relevance of these binding affinities.

### Ligand-Induced Thermodynamic Profiling: Characterization of *Pf*Ddi1-Ligand Complexes

**Fig. 6:**
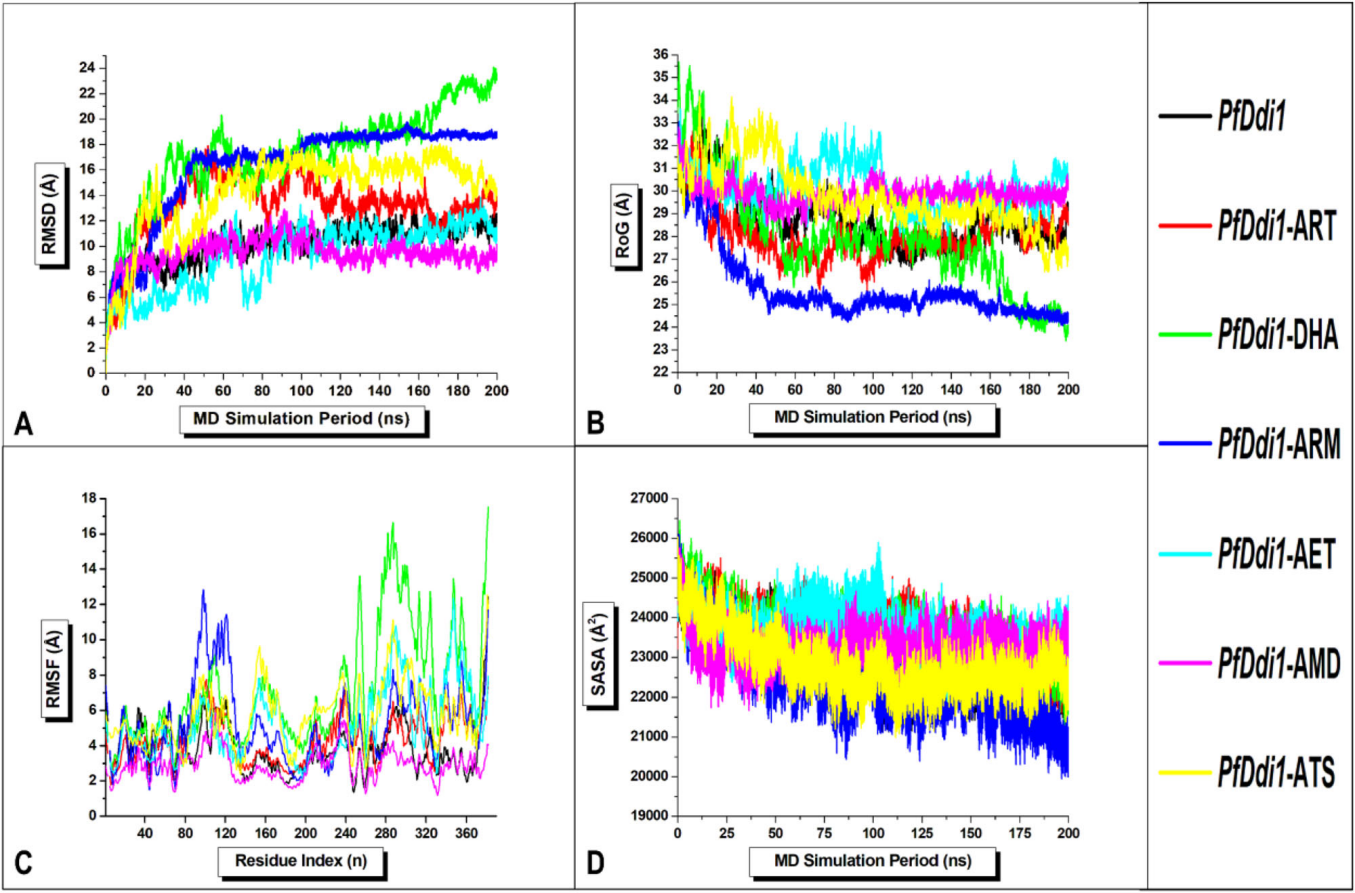
Visual graphical representation of **A** RMSD, **B** RoG, **C** RMSF, and **D** SASA computations of the c-α atoms of free (apo) *Pf*Ddi1 (black), *Pf*Ddi1-ART (red), *Pf*Ddi1-DHA (green), *Pf*Ddi1-ARM (blue), *Pf*Ddi1-AET (cyan), *Pf*Ddi1-AMD (pink), and *Pf*Ddi1-ATS (yellow) systems recorded across 200 ns MD simulations

Understanding the molecular behavior of *Pf*Ddi1 when subjected to a drug inhibitor forms the basis of drug development. It is worth noting that very little is known about conformational changes in *Pf*Ddi1 upon binding with artemisinin. Consequently, we conducted a comparative analysis, including RMSD, RoG, RMSF, and SASA, following molecular dynamics simulations over 200 ns [42, 43]. For each analysis, we juxtaposed the results obtained for all six *Pf*Ddi1 complex systems with those obtained for the free *Pf*Ddi1 system (apo). We assessed the structural stability of all systems by calculating the root mean square deviations (RMSD) of the c-α atoms as they reached convergence/deviation over the simulated time frame. A high RMSD value indicates a highly unstable system, whereas a low RMSD value signifies a highly stable system. The mean RMSD of *Pf*Ddi1, *Pf*Ddi1-ART, *Pf*Ddi1-DHA, *Pf*Ddi1-ARM, *Pf*Ddi1-AET, *Pf*Ddi1-AMD, and *Pf*Ddi1-ATS was 10.19, 13.21, 17.31, 16.36, 9.28, 9.49, and 14.33 Å, respectively. The highest RMSD value, in a decreasing order was recorded for *Pf*Ddi1-DHA, *Pf*Ddi1-ARM, *Pf*Ddi1-ATS, *Pf*Ddi1-ART, *Pf*Ddi1-AET, *Pf*Ddi1, and *Pf*Ddi1-ATS at 200, 200, 100, 140, 190, 200, and 17 ns, respectively. All systems appeared to have reached convergence by the 60 ns time step, with significant perturbations until 200 ns, except for *Pf*Ddi1-ARM, which showed marginal perturbations (Fig. 6A). Results from RMSD computations indicates that DHA, ARM, ATS, and ART decreased the structural stability of PfDdi1 in the order of decreasing strength, whereas AET increased its structural stability compared to AMD. Notably, *Pf*Ddi1 assumes a structurally unstable conformation, which is further exacerbated by ART and its derivatives.

In addition, the compactness of the systems was assessed by computing the radius of gyration (RoG) of the c-α atoms around the center of gravity as a measure of their rigidity over the entire simulation period. A high RoG value signifies a less rigid protein system, while a low value signifies a more rigid protein system. The mean RoG value of *Pf*Ddi1, *Pf*Ddi1-ART, *Pf*Ddi1-DHA, *Pf*Ddi1-ARM, *Pf*Ddi1-AET, *Pf*Ddi1-AMD, and *Pf*Ddi1-ATS was 28.77, 28.10, 27.94, 25.68, 30.22, 29.88, and 29.76 Å, respectively. Notably, DHA caused the highest and lowest RoG values of 35.69 and 23.40 Å at 7 and 200 ns, respectively. Among the systems, *Pf*Ddi1-ARM exhibited the least fluctuations, whereas the other systems, particularly *Pf*Ddi1-DHA, displayed significant fluctuations (Fig. 6B). Interestingly, ATS, AMD, and AET decreased the structural compactness of *Pf*Ddi1 in an increasing order of strength, whereas ARM, DHA, and ART increased it in a decreasing order of strength. These findings suggest that *Pf*Ddi1 adopts a more rigid conformation when bound to ARM than to AET, and that ART only reduces the rigidity of *Pf*Ddi1 by 0.67 Å.

Additionally, we assessed the structural flexibility of the systems by calculating the root mean square fluctuation (RMSF) of the c-α atoms as a measure of their relative displacement throughout the entire molecular dynamics simulation period. A high RMSF value indicates increased flexibility of the residues and a corresponding decrease in kinetic stability, whereas a low value indicates the opposite. The average RMSF value for *Pf*Ddi1, *Pf*Ddi1-ART, *Pf*Ddi1-DHA, *Pf*Ddi1-ARM, *Pf*Ddi1-AET, *Pf*Ddi1-AMD, and *Pf*Ddi1-ATS was 3.88, 4.31, 7.09, 5.24, 5.18, 2.90, and 5.77 Å, respectively. Except for AMD, all other ligand inhibitors, particularly DHA, reduced the overall kinetic stability of *Pf*Ddi1 (Fig. 6C). In comparison, the RVP domain exhibited greater residue flexibility than the UBL domain because of its increased binding potential with inhibitor drugs. Notably, ART, ARM, AET, ATS, and DHA increased the residue flexibility of the RVP domain in descending order of strength, whereas AMD reduced it, thus preserving the kinetic stability of the *Pf*Ddi1 catalytic domain. Conversely, ATS slightly increased the residue flexibility of the UBL domain compared to DHA, whereas AMD, ART, ARM, and AET decreased it in descending order of strength. Therefore, we can infer that the binding of ART and its derivatives to *Pf*Ddi1 may block the cleavage of ubiquitinated proteins by *Pf*Ddi1, leading to the accumulation of polyubiquitinated proteins and ultimately the death of the parasite.

Finally, we evaluated the exposure of *Pf*Ddi1’s hydrophobic residues to the solvent by calculating the solvent-accessible surface area (SASA) of c-α atoms. This analysis provides insight into protein complex folding and unfolding potential, as well as its solvent behavior over the simulated time step. In this case, a high SASA value indicates reduced hydrophobic stability and vice versa. The mean SASA value for *Pf*Ddi1, *Pf*Ddi1-ART, *Pf*Ddi1-DHA, *Pf*Ddi1-ARM, *Pf*Ddi1-AET, *Pf*Ddi1-AMD, and *Pf*Ddi1-ATS was 22980.89 Å^2^, 23545.02 Å^2^, 23494.95 Å^2^, 22317.68 Å^2^, 23691.12 Å^2^, 23212.86 Å^2^, and 22838.38 Å^2^, respectively. All the systems showed a comparable degree of solvent exposure across the entire 200 ns time step (Fig. 6D). Notably, the highest SASA value in order of strength was recorded for *Pf*Ddi1-ART (25567.48 Å^2^), *Pf*Ddi1 (25603.07 Å^2^), *Pf*Ddi1-AMD (25783.68 Å^2^), *Pf*Ddi1-AET (25895.91 Å^2^), *Pf*Ddi1-ATS (26025.95 Å^2^), *Pf*Ddi1-ARM (26099.04 Å^2^), and *Pf*Ddi1-DHA (26451.41 Å^2^) at 2, 1, 1, 100, 1, 1, and 1 ns, respectively. The SASA results indicated that ARM increased the hydrophobic stability of *Pf*Ddi1 compared to ATS, whereas AMD, DHA, ART, and AET reduced it in an increasing order of strength. Therefore, while ARM increases *Pf*Ddi1’s folding potential, AET increases its unfolding potential. The results obtained corroborate the findings of the RoG computations (Fig. 6B). Taken together, the aforementioned results suggest that ART and its derivatives confer varied conformational changes in *Pf*Ddi1, highlighting their ability to perturb the protein’s physiological functions and warrant their use as potential RVP inhibitor drugs.

### Principal Component Analysis (PCA)

**Fig. 7:**
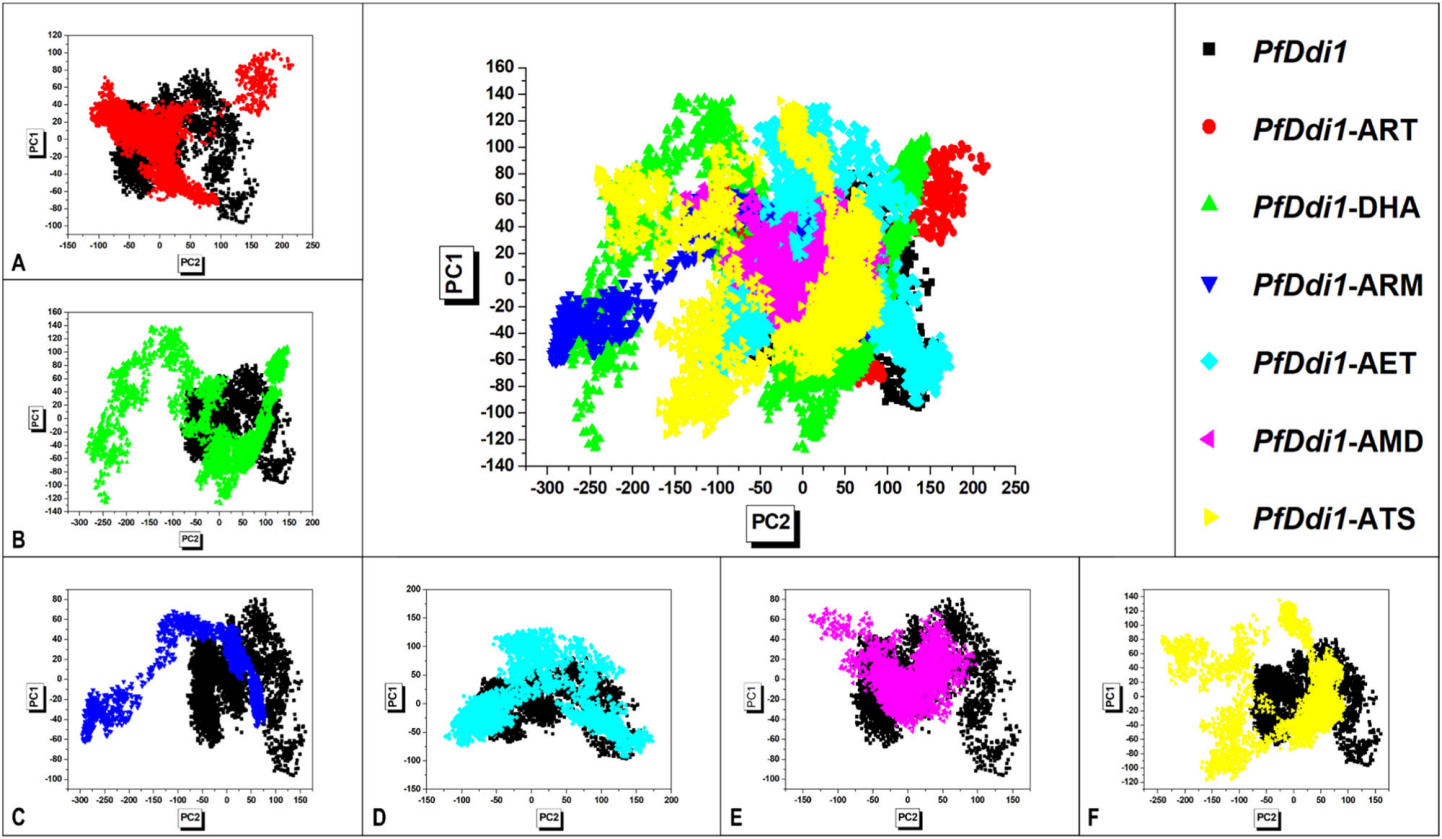
Visual graphical representation of PCA clustering showing the relative degree of c-α residue displacement of free (apo) *Pf*Ddi1 (black) relative to **A** *Pf*Ddi1-ART (red), **B** *Pf*Ddi1-DHA (green), **C** *Pf*Ddi1-ARM (blue), **D** *Pf*Ddi1-AET (cyan), **E** *Pf*Ddi1-AMD (pink), and **F** *Pf*Ddi1-ATS (yellow) systems recorded across 200 ns MD simulations

We investigated the degree of displacement of c-α atoms in *Pf*Ddi1 using PCA as a measure of the conformational dynamics induced by ART and its derivatives over a 200 ns simulation. Our analysis focused on the first two atomic motions (PC1 and PC2) along eigenvectors 1 (ev1) and 2 (ev2), respectively, in eigendecomposition of the covariance matrix [42]. Our results revealed that atomic motions were significantly dispersed along PC2 in *Pf*Ddi1-DHA compared to *Pf*Ddi1-ARM, *Pf*Ddi1-ATS, *Pf*Ddi1-ART, *Pf*Ddi1-AET, *Pf*Ddi1, and *Pf*Ddi1-AMD, in decreasing order of strength. Conversely, atomic motions captured along PC1 were significantly dispersed in *Pf*Ddi1-DHA compared to *Pf*Ddi1-ATS, *Pf*Ddi1-AET, *Pf*Ddi1-ART, *Pf*Ddi1, *Pf*Ddi1-ARM, and *Pf*Ddi1-AMD, in decreasing order of strength (Fig. 7). Notably, atomic motions in *Pf*Ddi1 were found to be unilateral in the presence of ARM, DHA, and ATS, but bilateral in the presence of AMD, AET, and ART. Furthermore, the aforementioned inhibitor drugs induced a denser cluster of atomic coordinates than the latter, which induced a more stable conformational state in *Pf*Ddi1. Overall, atomic motions were least restricted in PfDdi1 by DHA but were most restricted by AMD. The results obtained corroborate the findings of the RMSF computations (Fig. 6C). These findings shed light on the allosteric behavior of ART and its derivatives in *Pf*Ddi1.

The PCA plot (Fig. 7) visually highlights distinct differences across the various systems. For instance, the projections for *Pf*Ddi1 alone (black dots) and the *Pf*Ddi1-ligand complexes (colored dots) are spatially distinct, indicating clear variations in atomic motion. This indicates that each ligand induces unique conformational changes in *Pf*Ddi1, shifting its overall dynamics in a ligand-specific manner. Specifically, *Pf*Ddi1-ART (red) and *Pf*Ddi1-DHA (green) display broader distributions along PC1, reflecting increased flexibility and dynamic motion upon ligand binding. Conversely, *Pf*Ddi1-ARM (blue) and *Pf*Ddi1-AET (cyan) exhibit more localized clustering, suggesting that these ligands restrict the protein to a more constrained conformational space. Similarly, *Pf*Ddi1-AMD (pink) and *Pf*Ddi1-ATS (yellow) exhibit moderate distribution spreads that are distinct from each other, further underscoring their differential effects on residue motion. Furthermore, the eigenvectors corresponding to the highest eigenvalues indicate that different residues contribute most significantly to the principal motions for each system. This aligns with the Root Mean Square Fluctuation (RMSF) data, which shows varying residue flexibility patterns depending on the bound ligand. For instance, certain ligands, such as DHA and ART, enhance atomic displacement, which is indicative of increased flexibility, whereas others, such as ARM and AMD, stabilize specific structural motifs. Although some overlap exists owing to the shared structural features of *Pf*Ddi1, the unique clustering trends in the PCA confirm that the conformational behavior is not identical across all systems. This reinforces the idea that each ligand induces unique allosteric effects on *Pf*Ddi1, influencing its motion differently.

### Dynamic Cross Correlation Matrix (DCCM)

**Fig. 8:**
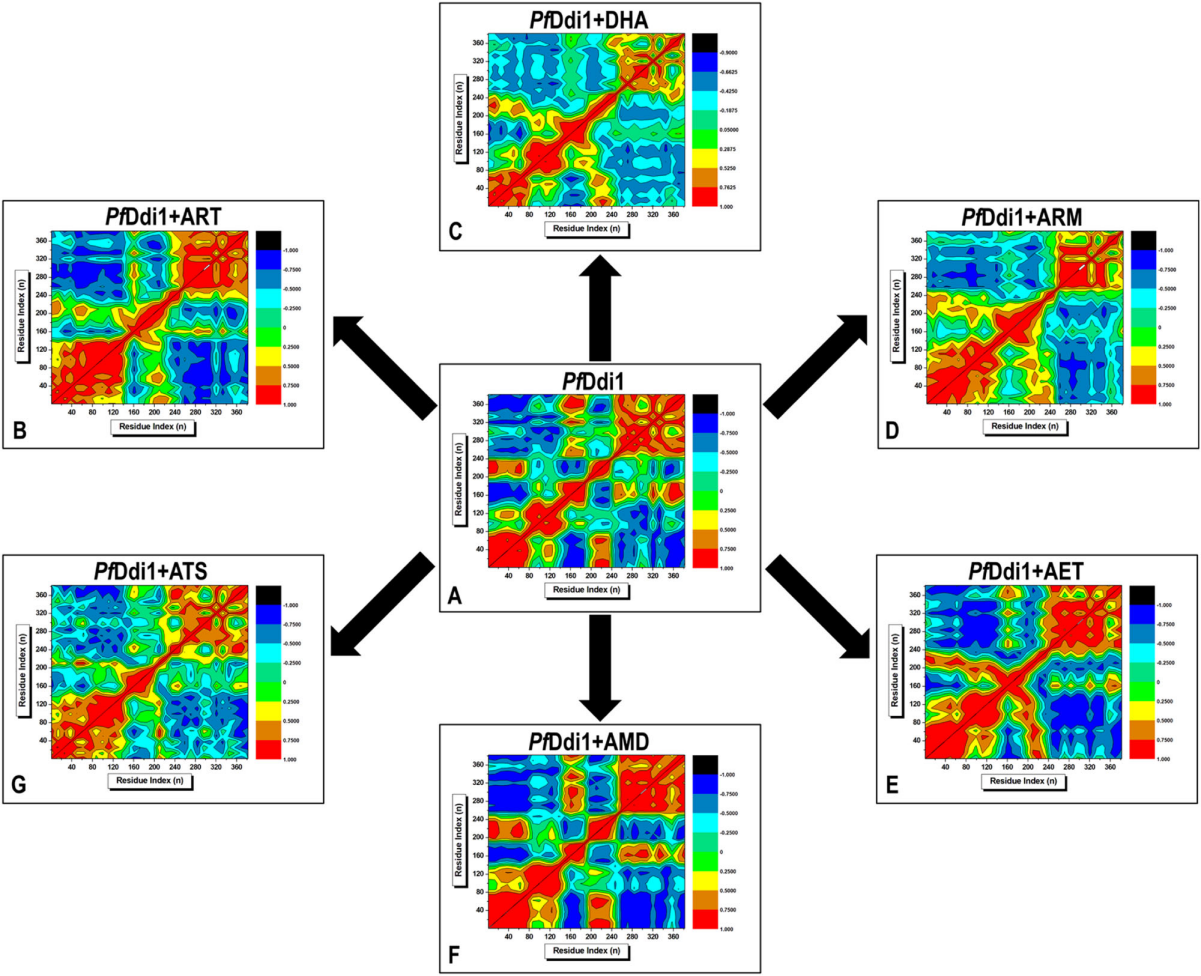
Visual graphical representation of DCCM showing the relative degree of c-α residue correlated/anti-correlated motion of **A** free (apo) *Pf*Ddi1 relative to **B** *Pf*Ddi1-ART, **C** *Pf*Ddi1-DHA, **D** *Pf*Ddi1-ARM, **E** *Pf*Ddi1-AET, **F** *Pf*Ddi1-AMD, and **G** *Pf*Ddi1-ATS systems captured across 200 ns MD simulations. The colored contour scale on the right of each graph shows the increasing strength of the anti-correlated motion from black to red

To better understand the nature of the relative motions of c-α atoms in *Pf*Ddi1, and to determine whether they moved in a correlated or non-correlated manner as a result of the binding effects of ART and its derivatives over a 200 ns time step, we utilized DCCM analysis. DCCM is a measurement tool that ranges from −1 (completely negative), 0 (no correlation), or +1 (completely positive) correlation of residue movement, and is represented by black to cyan, light green to green, and yellow to red contours, respectively [43]. Regions that exhibited positively correlated motions were indicative of protein function, as seen in both the UBL and RVP domains among the seven systems. In contrast, regions that exhibited negatively correlated motions suggested significant structural changes. Overall, *Pf*Ddi1 underwent the most inter-residue cooperative correlated motions in the presence of ARM. Additionally, the strength of inter-residue cooperative correlated motions was moderate in the presence of ATS compared to ART and DHA. In contrast, the highest degree of inter-residue negative non-cooperative correlated motion was observed in the presence of AET compared to AMD (Fig. 8). The results obtained corroborate the findings of the RoG and SASA computations (Fig. 6B, D). These findings highlight the diverse effects of inhibitors on the dynamic conformation of *Pf*Ddi1.

The observed positive correlation between the UBL and RVP domains is consistent with the high RMSD values observed for ligand-bound complexes, particularly *Pf*Ddi1-DHA and *Pf*Ddi1-ART. The high RMSD values suggest large-scale conformational rearrangements, which are reflected in the DCCM plots as an increase in interdomain-correlated motion over time. In contrast, *Pf*Ddi1-ARM and *Pf*Ddi1-AMD exhibit relatively lower RMSD values and weaker interdomain correlation, indicating that these ligands contribute to a more structurally stable complex. Moreover, Residue-level flexibility (RMSF analysis) highlights that regions contributing most to the positive correlation are predominantly in the loop regions connecting the UBL and RVP domains. Specifically, Loop 1 (GLN 266 - ILE 269) and Loop 2 (ILE 323 - TYR 326) show significant fluctuations, aligning with their role in interdomain communication. This suggests that the correlated movement between these two domains is largely influenced by loop flexibility, which varies depending on the bound ligand. Ligands such as DHA and ART cause an increase in loop flexibility, resulting in higher correlated motion, whereas ARM and AMD stabilize the structure, reducing flexibility and correlation. Additionally, the PCA results further reinforce these findings by illustrating how the principal motions of *Pf*Ddi1 vary across different ligand-bound systems. The clustering of PCA trajectories for *Pf*Ddi1-DHA and *Pf*Ddi1-ART shows a more widespread and dispersed conformational space, corresponding to their high interdomain correlation in DCCM and higher RMSD values. In contrast, *Pf*Ddi1-AMD and *Pf*Ddi1-ARM exhibit compact PCA clustering, suggesting a more restricted and stable conformational space, which aligns with their lower RMSD and weaker interdomain correlation.

### Free Binding Energy (FBE) Calculations

The binding free energy (BFE) between ART and its derivatives and *Pf*Ddi1 was determined using the MM/GBSA protocol over 50,000 MD snapshot frames. This allowed for the computation of individual energy components, providing insight into how each contributes to intermolecular association over time and predicts the binding affinity of an inhibitor drug [44]. The BFE values were measured in kcal/mol and were found to be −21.54 ± 0.12, −25.18 ± 0.20, −23.96 ± 0.14, −20.75 ± 0.18, −22.45 ± 0.16, and −34.24 ± 0.22 for *Pf*Ddi1-ART, *Pf*Ddi1-DHA, *Pf*Ddi1-ARM, *Pf*Ddi1-AET, *Pf*Ddi1-AMD, and *Pf*Ddi1-ATS, respectively. The results showed that ΔE_vdW_, ΔE_elec_, ΔG_GB_, and ΔG_SA_ contributed favorably to the stabilization of *Pf*Ddi1-ATS, *Pf*Ddi1-AMD, *Pf*Ddi1-AET, and *Pf*Ddi1-ATS, respectively, whereas ΔE_vdW_, ΔE_elec_, ΔG_GB_, and ΔG_SA_ contributed poorly to the stabilization of *Pf*Ddi1-ART, *Pf*Ddi1-AET, *Pf*Ddi1-AMD, and *Pf*Ddi1-ART, respectively (Table 3). These findings support the use of ART and its derivatives as potent inhibitors of *Pf*Ddi1, with ATS being the most ideal inhibitor drug and AET being the least ideal.

**Table 3:** Free binding energy terms expressed in kcal/mol with the standard error of mean of *Pf*Ddi1-ligand complexes. MM/GBSA was employed using 50,000 MD trajectory frames

*Pf*Ddi1-ligand Complex	Energy components (kcal/mol)						
	ΔE_vdW_	ΔE_elec_	ΔG_GB_	ΔG_SA_	ΔG_gas_	ΔG_solv_	ΔG_bind_
***Pf*Ddi1-ART**	−24.66 ± 0.13	−13.82 ± 0.20	20.04 ± 0.18	−3.10 ± 0.02	−38.48 ± 0.23	16.94 ± 0.18	−21.54 ± 0.12
***Pf*Ddi1-DHA**	−29.84 ± 0.15	−6.85 ± 0.21	15.07 ± 0.14	−3.57 ± 0.02	−36.69 ± 0.30	11.50 ± 0.13	−25.18 ± 0.20
***Pf*Ddi1-ARM**	−29.10 ± 0.14	−6.35 ± 0.12	14.82 ± 0.10	−3.32 ± 0.02	−35.46 ± 0.17	11.50 ± 0.11	−23.96 ± 0.14
***Pf*Ddi1-AET**	−25.91 ± 0.19	−4.72 ± 0.14	13.10 ± 0.15	−3.22 ± 0.02	−30.63 ± 0.27	9.88 ± 0.14	−20.75 ± 0.18
***Pf*Ddi1-AMD**	−27.22 ± 0.17	−119.22 ± 0.88	127.28 ± 0.93	−3.29 ± 0.02	−146.44 ± 0.96	123.99 ± 0.92	−22.45 ± 0.16
***Pf*Ddi1-ATS**	−39.99 ± 0.16	−29.78 ± 0.25	40.71 ± 0.22	−5.18 ± 0.02	−69.77 ± 0.35	35.53 ± 0.21	−34.24 ± 0.22

ΔE_elec_ (electrostatic energy), ΔE_vdW_ (van der Waals energy), ΔG_GB_ (polar solvation energy), ΔG_SA_ (non-polar solvation energy), ΔG_gas_ (gas-phase energy), ΔG_solv_ (Total solvation free energy of polar and non-polar states), and ΔG_bind_ (total free energy of binding)

The discrepancy between docking scores and MM/GBSA binding free energies (BFE) highlights the limitations of docking methods, such as AutoDock Vina, in capturing the full range of protein-ligand interactions. AutoDock Vina uses empirical scoring functions that may overlook important factors, such as solvation and entropy, while MM/GBSA integrates molecular mechanics and solvation models for a more detailed assessment. Docking methods sample fewer conformations and are based on static structures, whereas MM/GBSA evaluates dynamic interactions using molecular dynamics snapshots, providing a more accurate reflection of binding energies. The higher binding affinity observed for artesunate (ATS) in MM/GBSA, despite a comparable docking score to other ligands, demonstrates the importance of complementing docking studies with more advanced energy calculations, such as MM/GBSA, to capture realistic binding affinities.

### Per-Residue Energy Decomposition (PRED) Analysis

**Fig. 9:**
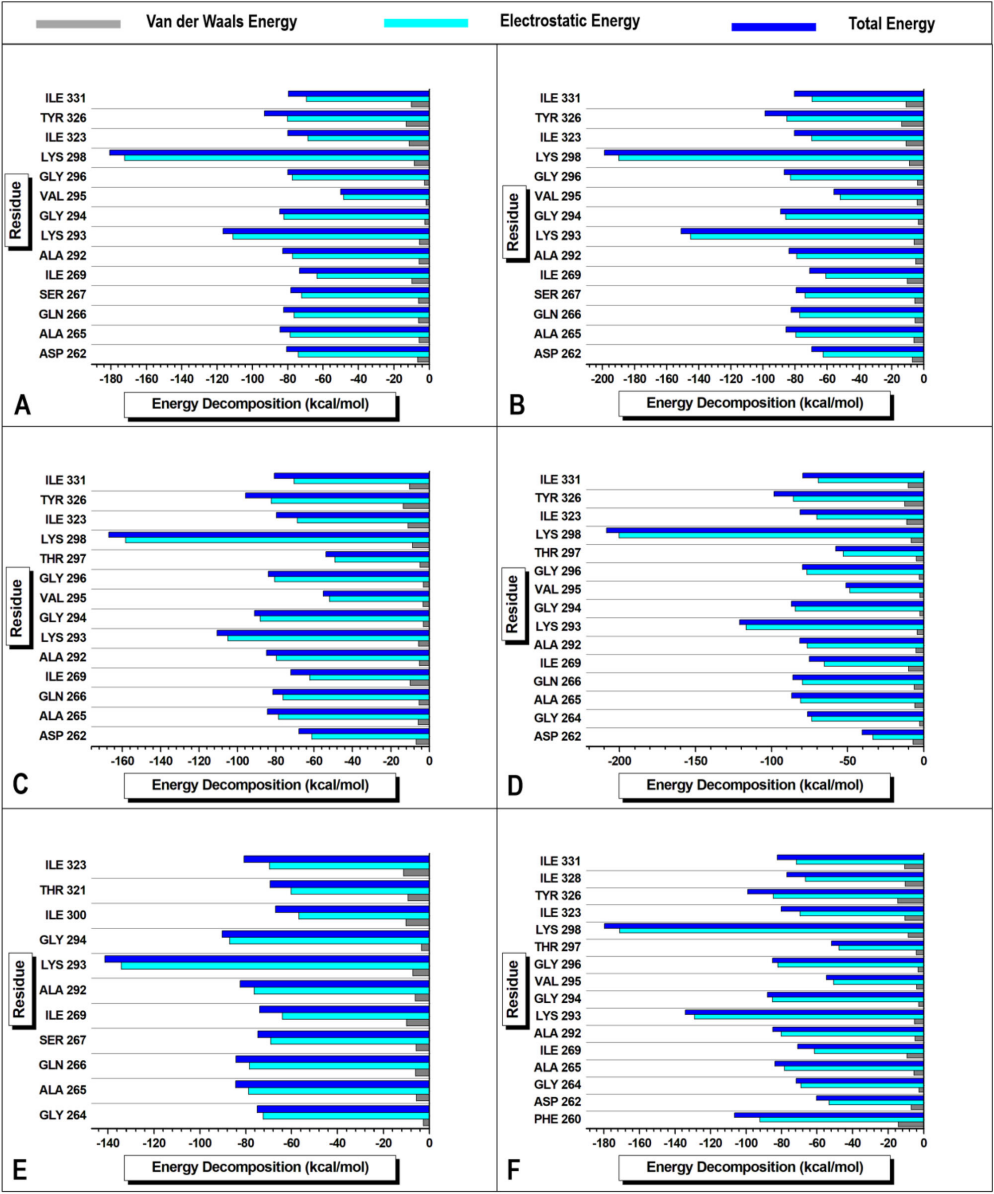
Visual graphical representation of PRED calculations showing the relative contribution of van der Waals (dark grey), electrostatic (cyan), and total (blue) total energy by *Pf*Ddi1 active-site residues in the complex with **A** ART, **B** DHA, **C** ARM, **D** AET, **E** AMD, and **F** ATS recorded across 50,000 MD snapshot trajectories

The electrostatic and van der Waals contributions of the active-site residues for each association between *Pf*Ddi1 and an inhibitor drug were analyzed using the BFE computed across 50,000 stabilized MD trajectory snapshots and the MM/GBSA protocol [43]. The total electrostatic energy contribution was found to be 12-, 12-, 11-, 13-, 11-, and 11-fold higher than the total van der Waals energy contribution in *Pf*Ddi1-ART, *Pf*Ddi1-DHA, *Pf*Ddi1-ARM, *Pf*Ddi1-AET, *Pf*Ddi1-AMD, and *Pf*Ddi1-ATS, respectively (Fig. 9). For the association between *Pf*Ddi1 and ART, LYS 298 and LYS 293, and ILE 269 and VAL 295 contributed the highest and lowest total energy contribution of −180.611 and −116.626 kcal/mol, and −73.334 and −50.123 kcal/mol, respectively. TYR 326 and ILE 323, and GLY 294 and VAL 295 made the largest and smallest van der Waals energy contributions of −13.116 and −11.32 kcal/mol, and −2.421 and −1.782 kcal/mol, respectively (Fig. 9A). For the association between *Pf*Ddi1 and DHA, LYS 298 and LYS 293, and ASP 262 and VAL 295 contributed the highest and lowest total energy contribution of −198.70 and −150.997 kcal/mol, and −69.554 and −55.855 kcal/mol, respectively. TYR 326 and ILE 331, and GLY 296 and GLY 294 made the largest and smallest van der Waals energy contributions of −13.703 and −10.995 kcal/mol, and −3.888 and −3.25 kcal/mol, respectively (Fig. 9B). For the *Pf*Ddi1-ARM association, LYS 298, LYS 293, VAL 295, and THR 297 contributed the highest and lowest total energy contributions of −167.037 and −110.584, and −55.176 and −53.859 kcal/mol, respectively. Additionally, TYR 326 and ILE 323 and GLY 296 and GLY 294 made the largest and smallest van der Waals energy contributions of −13.573 and −11.034, and −3.175 and −3.103 kcal/mol, respectively (Fig. 9C). The *Pf*Ddi1-AET association showed similar results, with LYS 298, LYS 293, VAL 295, and ASP 262 contributing the highest and lowest total energy contributions of −208.474 and −121.119, and −51.084 and −40.431 kcal/mol, respectively. Moreover, TYR 326 and ILE 323, and VAL 295 and GLY 294 made the largest and smallest van der Waals energy contributions of −12.683 and −11.174, and −2.597 and −2.505 kcal/mol, respectively (Fig. 9D). For the *Pf*Ddi1-AMD association, LYS 321 and ILE 300, and GLY 294 and GLY 264 contributed the highest and lowest total energy contributions of −141.253 and −90.307, and −69.356 and −66.894 kcal/mol, respectively. Additionally, ILE 323 and ILE 300, and GLY 294 and GLY 264 made the largest and smallest van der Waals energy contributions of −11.206 and −10.114, and −3.371 and −2.635 kcal/mol, respectively (Fig. 9E). Finally, for the *Pf*Ddi1-ATS association, LYS 298, LYS 293, VAL 295, and THR 297 contributed the highest and lowest total energy contributions of −179.736 and −134.201 and −54.667 and −51.847 kcal/mol, respectively. TYR 326 and PHE 260, and GLY 294 and GLY 264 had the largest and smallest van der Waals energy contributions of −14.571 and −14.305, and −2.795 and −2.624 kcal/mol, respectively (Fig. 9F).

### Active-Site Residue Interaction Framework Analysis

**Fig. 10:**
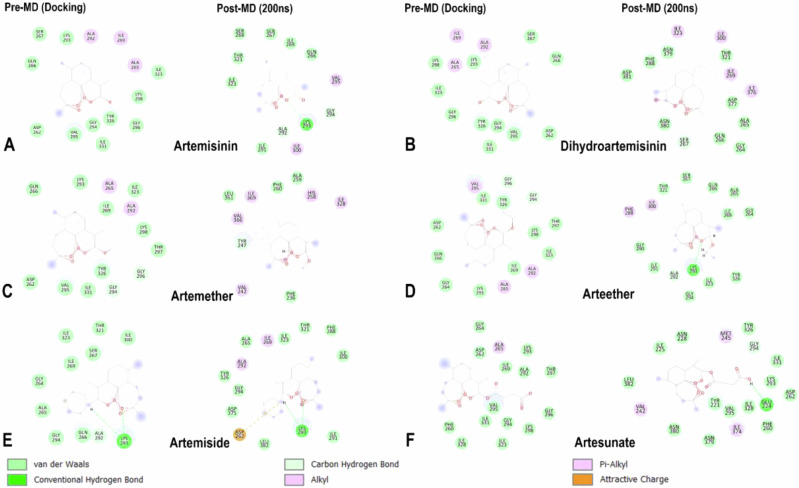
Visual representation of the active-site residue interaction framework of *Pf*Ddi1 complex with **A** ART, **B** DHA, **C** ARM, **D** AET, **E** AMD, and **F** ATS using pre-MD (docking) and post-MD (200 ns) structures. Legend shows the varied bond types

The study of the *Pf*Ddi1 intermolecular interaction framework with its associated ligands revealed insights into their structural and functional diversity, which could be useful for drug design research. In the *Pf*Ddi1-ART complex, alkyl interactions were observed between ALA 265, ILE 269, and ALA 292 with the methylcyclohexane core. Additionally, van der Waals interactions were identified for several residues, including ASP 262, VAL 295, GLY 294, ILE 331, TYR 326, GLY 296, LYS 298, ILE 323, LYS 293, SER 267, and GLN 266, as shown in Fig. 10A. Similarly, in the *Pf*Ddi1-DHA complex, alkyl interactions were observed between ALA 265, ILE 269, and ALA 292 with the methylcyclohexane core, while van der Waals interactions were identified for ASP 262, VAL 295, GLY 294, ILE 331, TYR 326, GLY 296, LYS 298, ILE 323, LYS 293, SER 267, and GLN 266 (Fig. 10B). In the *Pf*Ddi1-ARM complex, alkyl interactions were established between ALA 265 and ALA 292 with the methylcyclohexane core, and carbon hydrogen bonds were observed between GLY 294 and the 0 atom of methoxymethane of the 2-methoxy-3-methyloxane group, as well as between GLY 296 and the C atom of methanol of the same group. Additionally, van der Waals interactions were observed for a range of amino acids, including ASP 262, VAL 295, ILE 331, TYR 326, THR 297, LYS 298, ILE 269, ILE 323, LYS 293, and GLN 266, as depicted in Fig. 10C.

In the *Pf*Ddi1-AET complex, there were alkyl interactions between ALA 265 and ALA 292 with the methylcyclohexane core, as well as a bond between VAL 295 and the C atom of the 1-methyl-6,7,8-trioxabicyclo[3.2.2]nonane group. Additionally, carbon hydrogen bonds were observed between GLY 294 and the O atom of methoxyethane of the 2-ethoxy-3-methyloxane group, and between GLY 296 and the C atom of methanol of the same group group. Furthermore, there were van der Waals forces between ASP 262, GLN 266, GLY 264, LYS 293, ILE 269, ILE 323, LYS 298, THR 297, TYR 326, and ILE 331 (Fig. 10D). In the *Pf*Ddi1-AMD complex, LYS 293 formed hydrogen bonds with 03 of the 1-methyl-6,7,8-trioxabicyclo[3.2.2]nonane group, and with the protonated H atom of the thiomorpholin-4-ium group. ALA 292 formed a carbon hydrogen bond with 01 of the 1-methyl-6,7,8-trioxabicyclo[3.2.2]nonane group, and an alkyl bond with the methylcyclohexane core. van der Waals interactions were observed between ILE 300, THR 321, SER 267, ILE 269, ILE 323, GLY 264, ALA 265, GLY 294, and GLN 266 (Fig. 10E). In the *Pf*Ddi1-ATS complex, ALA 265 established an alkyl bond with the methylcyclohexane core. An unfavorable acceptor-acceptor clash was observed between TYR 326 and 03 of the 1-methyl-6,7,8-trioxabicyclo[3.2.2]nonane group, suggesting a potential destabilizing factor. Interactions of van der Waals energy were established between ASP 262, GLY 264, ILE 269, ALA 292, LYS 293, THR 297, GLY 296, LYS 298, GLY 294, ILE 323, ILE 331, VAL 295, ILE 328, and PHE 260 (Fig. 10F). Notably, the methylcyclohexane core of all the ligands maintained consistent alkyl interactions with ALA 265 and/or ALA 292, suggesting a consistent binding motif across the complexes. It is notable that artemiside (AMD) exhibits a lower binding affinity than ART and DHA, despite forming hydrogen bonds with LYS 293 (Fig. 10E). Unlike AMD, ART and DHA form alkyl hydrophobic interactions with residues such as ALA 265 and ALA 292, which may significantly contribute to stabilizing these ligands within the binding pocket of *Pf*Ddi1, thereby enhancing their overall binding affinity. This highlights the critical role of hydrophobic interactions in stabilizing ligand-protein complexes and underscores the need to consider these interactions when designing more effective inhibitors targeting *Pf*Ddi1.

Consequently, we investigated the interaction networks of the complexes after 200 ns of MD simulations, providing insights into the evolution of protein-ligand interactions and the dynamic nature of these systems. For the *Pf*Ddi1-ART complex, new carbon-hydrogen interactions were observed with ALA 292 and GLY 294, a conventional hydrogen bond with LYS 293, and alkyl interactions with VAL 295 and ILE 300. In the *Pf*Ddi1-DHA complex, multiple alkyl interactions were established with ILE 300, ILE 323, ILE 269, and ILE 376, along with a carbon-hydrogen bond with SER 267. In the *Pf*Ddi1-ARM complex, TYR 247 exhibited multiple π-alkyl interactions in addition to forming a carbon-hydrogen bond. Furthermore, a π-alkyl interaction with HIS 258 and alkyl bonds with VAL 366, ILE 369, VAL 242, and ILE 328 were also observed. For the *Pf*Ddi1-AET complex, ALA 292 formed new carbon-hydrogen and alkyl interactions, while LYS 293 engaged in both carbon-hydrogen and conventional hydrogen bonding, in addition to alkyl interactions. Furthermore, π-alkyl interactions with PHE 288 and alkyl interactions with ILE 300 were also identified. In the *Pf*Ddi1-AMD complex, an attractive electrostatic interaction was observed with ASP 262 along with alkyl interactions involving ALA 292 and ILE 269. LYS 293 retained its conventional hydrogen bond while forming an additional alkyl bond. Finally, in the *Pf*Ddi1-ATS complex, new alkyl bonds were formed with VAL 242, MET 245, and ILE 374, while carbon-hydrogen and conventional hydrogen bonds were observed with GLY 294 and GLU 224. Overall, the interaction networks between ART and its derivatives with *Pf*Ddi1 appears to be predominantly governed by hydrophobic interactions and van der Waals contacts, highlighting the critical role of these forces in stabilizing the complexes.

### Comparative Analysis of *PfD*di1-Ligand Interactions and Identification of Targetable Loops for Drug Design

**Fig. 11:**
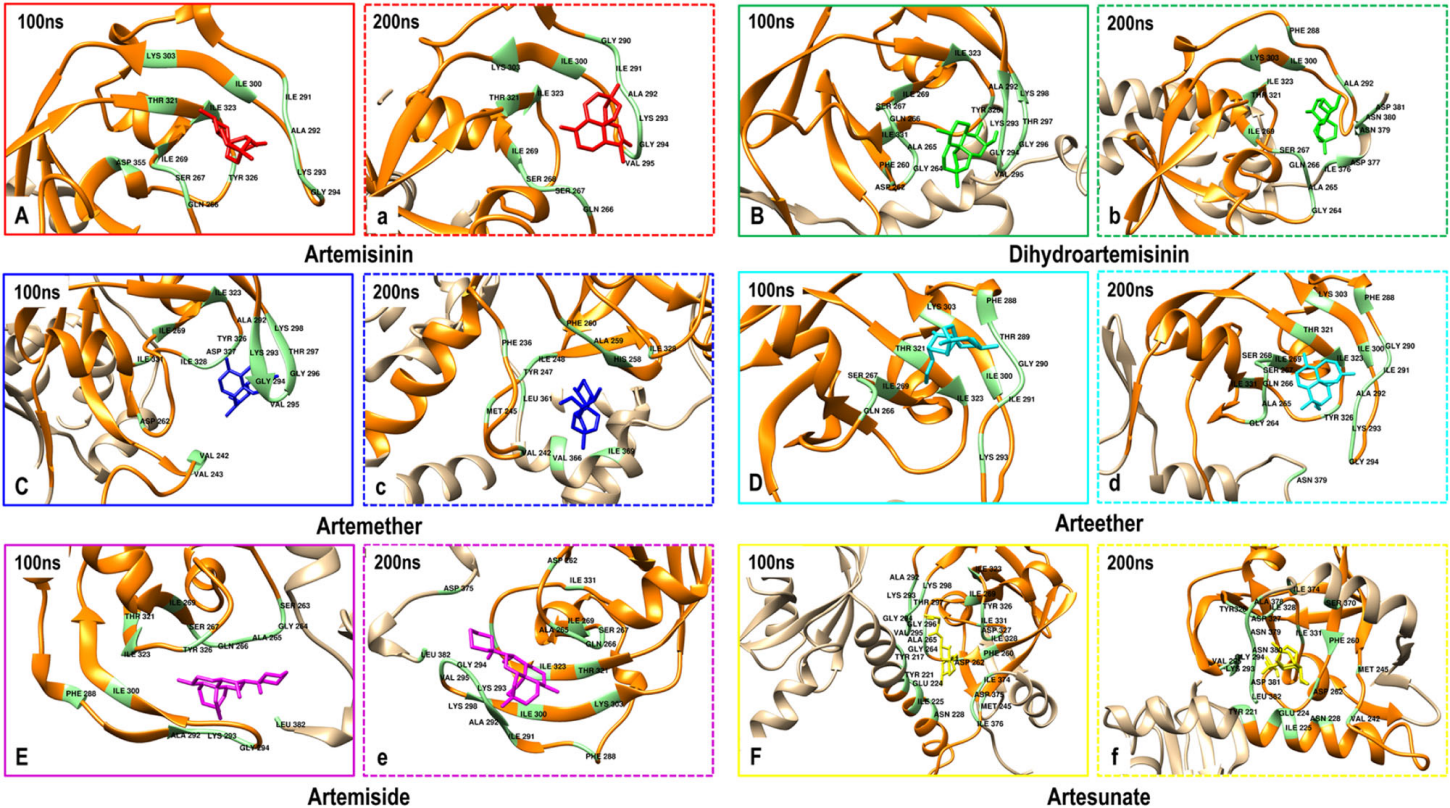
Visual representation of *Pf*Ddi1-ligand residue interaction network (light green) taken from 10,000 (solid line) and 20,000 (dashed line) MD trajectory snapshots of each *Pf*Ddi1 complex with (**A**, **a**) ART (red), (**B**, **b**) DHA (green), (**C**, **c**) ARM (blue), (**D**, **d**) AET (cyan), (**E**, **e**) AMD (pink), and (**F**, **f**) ATS (yellow). Residue interactions with the putative active-site RVP domain (orange) and the non-active-site domain (tan) are shown

Understanding the dynamics of the interaction between *Pf*Ddi1 and various ligands can provide valuable insights into the structural basis of ligand binding and identify the key residues that are critical for this process. This information can be used to design new therapeutic agents for malaria. To this end, we conducted a comparative analysis of the interactions between *Pf*Ddi1 and the six ligands (ART, DHA, ARM, AET, AMD, and ATS) using 100,000 and 200,000 trajectory snapshots. Our analysis identified common residues, such as GLN 266, SER 267, ILE 269, ILE 323, TYR 326, ALA 292, LYS 293, and GLY 294, which may play crucial roles in ligand binding. Additionally, we found that THR 321 interacted frequently in ART-, AET-, and AMD-*Pf*Ddi1 complexes, whereas ASP 262 was observed in DHA-, ARM-, and ATS- *Pf*Ddi1 complexes. Other residues such as PHE 288, ILE 300, and LYS 303 were found in several complexes, emphasizing their importance. Furthermore, unique residues, such as ASN 379, ASN 380, ASP 381, and LEU 382, were observed in the AMD-*Pf*Ddi1 and ATS-*Pf*Ddi1 complexes, suggesting specific binding interactions with these ligands. Notably, certain residues, such as HIS 258, ALA 259, PHE 260, ILE 248, TYR 247, LEU 361, PHE 236, MET 245, VAL 366, and ILE 369, were specific to the ARM-*Pf*Ddi1 interaction at 200 ns, indicating a distinct binding profile (Fig. 11).

Critical binding motifs were identified namely, Loop 1 (GLN 266 - ILE 269), Loop 2 (ILE 323 - TYR 326), and Loop 3 (ALA 292 - GLY 294) present in the interactions of ART-, DHA-, ARM-, AET-, AMD-, and ATS-*Pf*Ddi1 complexes. These regions are considered potential targets because they are consistently involved in interactions with all ligands, suggesting that they represent structurally flexible or exposed areas that facilitate ligand-binding. This makes them more accessible and vulnerable to small molecule inhibitors. Additionally, these loops contain residues that contribute to the stabilization of ligand complexes, reinforcing their importance as key interaction motifs (Fig. 9). Therefore, designing inhibitors that disrupt these motifs may impair the functionality of the protein, which is ideal for therapeutic interventions.

In addition, Loop 4 (SER 267 - ILE 269) was common in ART-, DHA-, AET-, AMD-, and ATS-*Pf*Ddi1 complexes, whereas Loop 5 (THR 321–ILE 323) was common in ART-, AET-, and AMD-*Pf*Ddi1 complexes. Subsequently, ligand-specific loops were identified for ARM (HIS 258-PHE 260) and both AMD and ATS (ASN 379-LEU 382). The analysis identified several key loops in *Pf*Ddi1 that are essential for ligand-binding. Loops, such as GLN 266 - ILE 269, ILE 323 - TYR 326, and ALA 292 - GLY 294 are critical for multiple ligand interactions, making them prime targets for broad-spectrum drug design. These unique loops and interactions specific to certain ligands provide opportunities for the development of selective inhibitors. This comprehensive understanding of the interaction framework can significantly aid the rational design of new therapeutic agents targeting *Pf*Ddi1.

## Conclusion

Research indicates that the RVP domain of *Plasmodium falciparum* could be a promising target for combating artemisinin resistance and for enhancing antimalarial treatments. Therefore, this study evaluated the binding affinity of artemisinin (ART) and its derivatives, DHA, ARM, AET, AMD, and ATS, towards *P. falciparum* DNA-damage-inducible protein 1 (*Pf*Ddi1) and analyzed its effects on its conformational dynamics through in silico modelling studies. All ligands displayed high binding affinity for the RVP domain, interacting with highly conserved residues. Thermodynamic profiling revealed that *Pf*Ddi1 is inherently structurally unstable, with ART and most derivatives potentially exacerbating this instability, except for AET and AMD. ARM increased *Pf*Ddi1 conformational rigidity the most, whereas AET decreased it the most. AMD enhanced its kinetic stability the most, whereas DHA decreased it the most. Hydrophobic stability was highest with ARM and lowest with AET. Atomic motions were most dispersed with DHA and least dispersed with AMD along the principal components. ARM caused the most positively correlated atomic motions, whereas AET caused the most negatively anti-correlated motions. These findings underscore the varied conformational effects of the ligands on *Pf*Ddi1.

Binding energy computations ranged from −20.75 ± 0.18 to −34.24 ± 0.22 kcal/mol, indicating the potency of ART and its derivatives as inhibitors of *Pf*Ddi1, with ATS showing the highest binding affinity. The electrostatic energy contributions were significantly higher than the van der Waals contributions in all *Pf*Ddi1-ligand complexes. LYS 298 and LYS 293 were the primary contributors to the binding energy in most complexes, while ILE 300 and LYS 321 were significant in the *Pf*Ddi1-AMD complex. A consistent alkyl interaction with ALA 265 and/or ALA 292 by the methylcyclohexane core of all ligands highlighted a common binding motif. Comparative analysis identified critical binding motifs, Loop 1 (GLN 266 - ILE 269), Loop 2 (ILE 323 - TYR 326), and Loop 3 (ALA 292 - GLY 294), which could be pivotal for multiple ligand interactions and thus serve as targets for broad-spectrum drug design. Ligand-specific interactions were noted in Loop 4 (SER 267 - ILE 269) and Loop 5 (THR 321 - ILE 323), offering pathways for selective inhibitor development.

While AlphaFold generates static 3D structures of proteins based on their amino acid sequences, the generated models do not account for the dynamic nature of proteins, especially for highly flexible proteins such as *Pf*Ddi1. In addition, molecular simulations may not fully capture the complexity of protein conformational dynamics in biological contexts. Furthermore, the absence of experimental structural data for *Pf*Ddi1 limits the extent to which the computational findings can be validated. Overall, this study highlights the inhibitory potential of ART and its derivatives on *Pf*Ddi1 from an atomistic standpoint, necessitating experimental validation.

## Supplementary information

Supplementary Material

## Data Availability

The authors confirm that data supporting the findings of this study are included in this paper. If additional data files are required in a different format, they may be obtained from the corresponding author upon reasonable request.
