# Supplementary material for: Computational Analysis of Plasmodium falciparum DNA Damage Inducible Protein 1 (PfDdi1): Insights into Binding of Artemisinin and its Derivatives and Implications for Antimalarial Drug Design
Source: Cell Biochem Biophys. 2025 Mar 20;83(3):3277–97. doi: 10.1007/s12013-025-01709-2 (PMC12414056; doi:10.1007/s12013-025-01709-2)
Supplement: Supplementary file 1 — Supplementary Material [file 12013_2025_1709_MOESM1_ESM.docx]

**Computational Analysis of *Plasmodium falciparum* DNA Damage Inducible Protein 1 (*Pf*Ddi1): Insights into Binding of Artemisinin and Its Derivatives and Implications for Antimalarial Drug Design**

Ernest Oduro-Kwateng^1^, Ibrahim Oluwatobi Kehinde^1^, Musab Ali^1^, Kabange Kasumbwe^2^, Vuyisa Mzozoyana^3^, Narasimham L. Parinandi^4^, and Mahmoud E. S. Soliman^1*^

**Table S1**: Docking scores of ART and its derivatives from triplicate docking calculations.

| **Ligand** | **Score 1** | **Score 2** | **Score 3** |
| --- | --- | --- | --- |
| ART | -7.2 | -7.2 | -7.2 |
| DHA | -7.4 | -7.4 | -7.4 |
| ARM | -6.4 | -6.4 | -6.4 |
| AET | -6.4 | -6.4 | -6.4 |
| AMD | -6.8 | -6.8 | -6.8 |
| ATS | -7 | -7 | -7 |

**Table S2**: Summary statistics of docking scores for artemisinin and derivatives on *Pf*Ddi1 active site.

| **Ligand** | **Count** | **Sum** | **Average** | **Variance** |
| --- | --- | --- | --- | --- |
| ART | 3 | -21.6 | -7.2 | 0 |
| DHA | 3 | -22.2 | -7.4 | 1.18x10^-30^ |
| ARM | 3 | -19.2 | -6.4 | 1.18x10^-30^ |
| AET | 3 | -19.2 | -6.4 | 1.18x10^-30^ |
| AMD | 3 | -20.4 | -6.8 | 0 |
| ATS | 3 | -21 | -7 | 0 |

**Table S3**: ANOVA results for binding affinities of artemisinin and derivatives.

| **Source of Variation** | **SS** | **df** | **MS** | **F** | **p-value** | **F crit** |
| --- | --- | --- | --- | --- | --- | --- |
| Between Groups | 2.56 | 5 | 0.512 | 8.65383x10^29^ | 6.6718x10^-177^ | 3.105875239 |
| Within Groups | 7.09975x10^-30^ | 12 | 5.91646x10^-31^ |  |  |  |
| Total | 2.56 | 17 |  |  |  |  |

**Table S4**: Tukey’s Post-Hoc test results for pairwise comparisons of docking scores.

| **Comparison** | **Mean Difference** | **Standard Error** | **q-Value** | **p-Value** | **Significant (Yes/No)** |
| --- | --- | --- | --- | --- | --- |
| DHA vs ART | 0.2 | 0.03968 | 5.033 | 0.0015 | Yes |
| ARM vs ART | 0.8 | 0.03968 | 20.135 | <0.001 | Yes |
| AET vs ART | 0.8 | 0.03968 | 20.135 | <0.001 | Yes |
| AMD vs ART | 0.4 | 0.03968 | 10.068 | <0.001 | Yes |
| ATS vs ART | 0.2 | 0.03968 | 5.033 | 0.0015 | Yes |
| DHA vs ARM | 1 | 0.03968 | 25.169 | <0.001 | Yes |
| DHA vs AET | 1 | 0.03968 | 25.169 | <0.001 | Yes |
| DHA vs AMD | 0.6 | 0.03968 | 15.101 | <0.001 | Yes |
| DHA vs ATS | 0.4 | 0.03968 | 10.068 | <0.001 | Yes |
| ARM vs AET | 0 | 0.03968 | 0 | 1 | No |
| ARM vs AMD | 0.4 | 0.03968 | 10.068 | <0.001 | Yes |
| ARM vs ATS | 0.2 | 0.03968 | 5.033 | 0.0015 | Yes |
| AET vs AMD | 0.4 | 0.03968 | 10.068 | <0.001 | Yes |
| AET vs ATS | 0.2 | 0.03968 | 5.033 | 0.0015 | Yes |
| AMD vs ATS | 0.2 | 0.03968 | 5.033 | 0.0015 | Yes |
